# Sequential mediation of early temperament and eating behaviors in the pathways from feeding practices to childhood overweight and obesity

**DOI:** 10.3389/fpubh.2023.1122645

**Published:** 2023-09-11

**Authors:** Xiaoning Zhang, Qiong Zhou, Nathaniel Kossi Vivor, Wei Liu, Junli Cao, Sheng Wang

**Affiliations:** ^1^School of Nursing, Hangzhou Normal University, Hangzhou, Zhejiang, China; ^2^Zhejiang Philosophy and Social Science Laboratory for Research in Early Development and Childcare, Hangzhou Normal University, Hangzhou, Zhejiang, China; ^3^School of Anesthesiology, Xuzhou Medical University, Xuzhou, China; ^4^Department of Medical Nursing, Union Technical Institute, Lianyungang Subbranch of Traditional Chinese Medicine, Lianyungang, Jiangsu, China; ^5^School of Management, Xuzhou Medical University, Xuzhou, China

**Keywords:** feeding practices, temperament, eating behaviors, overweight and obesity, infant and young child

## Abstract

**Introduction:**

Childhood eating behaviors and temperament may have important implication for constructing the pathways from maternal feeding practices to childhood overweight and obesity (OW/OB). Examining multiple feeding styles simultaneously to childhood OW/OB is critical through the mediators of early childhood temperament and eating behaviors.

**Methods:**

This cross-sectional study recruited mothers mainly responsible for child care from two hospitals and two healthcare centers in eastern China. Sociodemographic characteristics, and data from the Infant Feeding Style Questionnaire (IFSQ), the short form of Children Behavior Questionnaire [Revised (IBQ-RSF)], and the Child Eating Behavior Questionnaire for toddler (CEBQ-T) were collected. Weight and recumbent length were measured to calculate the age- and sex-specific body mass index (BMI) z-scores (BMIz). The structural equation modeling (SEM) approach was used to examine direct and indirect pathways from five maternal feeding styles to childhood OW/OB through temperament and eating behaviors.

**Results:**

A total of 486 children were recruited, 73 (15.02%) children were OW/OB; the age of the children was 14.55 (SD = 5.14) months, and the age of the mothers was 29.90 (SD = 3.63) years. The responsive feeding exerted significant direct (*β* = −0.098), indirect (*β* = −0.136) and total (*β* = −0.234) effects on childhood OW/OB. Restrictive feeding had significant direct (*β* = 0.222), indirect (*β* = 0.102) and total (*β* = 0.324) effects on childhood OW/OB. Indulgent feeding had significant direct (*β* = 0.220), indirect (*β* = 0.063), and total (*β* = 0.283) effects on childhood OW/OB. Pressuring feeding had significant direct (*β* = −0.116), indirect (*β* = −0.096) and total (*β* = −0.212) effects on childhood OW/OB.

**Discussion:**

There was a direct effect of feeding practices on childhood OW/OB; feeding practices indirectly predicted childhood OW/OB through temperament and eating behaviors in children aged 6–23 months. This study could help governments agencies, policymakers, and healthcare workers to establish optimal intervention programs targeting feeding practices through childhood eating behaviors and temperament to prevent childhood OW/OB.

## Background

Infancy and young childhood (0–24 months) are critical periods for developmental plasticity, with long-lasting behavioral consequences and may potential influence long-term risk of obesity (1). During this time, eating behaviors develop as children transition from exclusive milk to a modified adult diet, including main meals and snacks, to achieve stability over time (2). Complementary feeding (CF) introduces solid and semi-solid foods to satisfy nutritional requirements of children aged 6–23 months (3). Developmental theories suggest that parent–child interaction through feeding practices may influence child health outcomes (4). Recently, global approaches to feeding practices have emerged to address childhood overweight and obesity (OW/OB) (5). Feeding practice include parental beliefs and behaviors concerning feeding, as well as interactions with children in relation to food and food-related parenting (6). Feeding practices are key factors influencing eating behaviors through the quantity and quality of food provisions and the interactions around feeding, as infants learn to eat and parents shape the physical and social environment of eating (2). Infant and young child feeding practices (IYCF) (6–23 months) have been identified as an under-prioritized strategy to prevent childhood OW/OB (3), which include the following: (1) responsive parents are attentive to the satiety and hunger cues of their children and monitor the dietary quality; (2) restrictive parents limit the quantity and quality of unhealthy foods; (3) pressuring parents are concerned about the increasing amount of food, pressuring children to finish bottle or plate, or soothing children with food instrumentally; (4) indulgent parents do not limit the quantity or quality of food; and (5) laissez-faire parents do not limit dietary quality or quantity, with little interaction (7).

Study on feeding practices indicate that parents socialize with children to create an emotional climate around eating, and individual differences should be considered in the parent–child feeding process (8). Temperament refers to constitutionally based individual differences in the reactivity and self-regulation aspects of behavior (4), which is a long-standing research area in emotional development and well-being (9). Childhood characteristics can be approached from a broader perspective through temperament, a range of direct relationships between temperament and weight status in predicting childhood OW/OB has been reported (10). Current conceptualizations of childhood temperament have gained research attention, and the early emerging basic dispositions in three domains (11): (1) Surgency/Extraversion refers to the level of impulsivity, activity, sensation seeking, and positive anticipation; (2) Negative affectivity encompasses the propensity to experience emotions, e.g., frustration, fear, sadness, and anger; (3) Effortful control refers to the infant’s ability to inhibit behavioral responses to stimuli, concentrate and shift attention, exhibit perceptual sensitivity, have a lower threshold for experiencing pleasure, and demonstrate soothability, including the capacity for orientation and regulation in various aspects of the infant’s behavior and responses (12). There is evidence of moderate temperament stability from infancy to young childhood, and the assumption linking temperament in early childhood to later behavioral problems has been extensively documented (13). In response to childhood temperament, feeding practices have the potential to change (14), temperament may indirectly affect weight by prompting specific responses from maternal feeding practices; higher negative levels of temperament are associated with weight gain (15).

With the growth of the Chinese economy, there has been a significant increase in people migrating away from their hometowns. A commonly heard saying, “happiness is the taste of mom (in childhood),” implies that maternal feeding practices may serve as an additional predictor of eating behaviors. Feeding and eating in East Asian cultures has unique significance, previous studies showed that Chinese mothers reported higher responsive feeding and lower indulgent feeding (3, 7). Eating behaviors among young children include two traits: food approach and food avoidance. Food approach is characterized by a higher avid appetite and interest in food, and it has been associated with higher childhood weight, including the following: (1) food responsiveness (FR) involves assessing the extent to which children are demanding when being fed and how responsive they are to external food cues, e.g., children would be fed when they see or smell food; (2) enjoyment of food (EF) captures the perceived pleasure childhood experience from food and the general feeding process, e.g., the extent to which children favor and derive satisfaction from eating; and (3) emotional overeating (EOE) captures the tendency of children to eat more in response to stress and negative emotions. Food avoidance is characterized by lower appetite and interest in food and has been associated with lower weight, including the following: (1) food fussiness (FF) measures the tendency of children to be highly selective in accepting the texture and flavor of foods, including a reluctance to try new food; (2) satiety responsiveness (SR) measures the satiety sensitivity to internal cues of children, e.g., how easily children become full around mealtime; and (3) slowness in eating refers to the measurement of the speed at which children consume typical food, e.g., the overall feeding pace during childhood (16).

Feeding practices influence childhood eating behaviors during processes by communicating of behaviors and beliefs about food and eating (17). Eating behaviors can contribute to poor nutritional status among children (18), and are associated with childhood OW/OB (19). Parents directly control childhood food intake as they may pressure children to eat more or restrict children their consumption of unhealthy foods, controlling feeding may be exerted indirectly by monitoring the intake of unhealthy foods in children (17). Study has found associations between parental food restriction and childhood food approach behaviors and OW/OB, e.g., enjoyment of food and food responsiveness (20). Childhood food-avoidant behaviors, e.g., food fussiness and satiety responsiveness, are also associated with pressure feeding and lower childhood weight status (21). While higher parental food restriction is associated with a higher childhood OW/OB, higher pressuring feeding is associated with higher childhood underweight (22), which in turn may influence childhood lower interest in food (23) or the development of obesogenic eating behaviors (24).

External feeding practices have been found to be associated with the development of childhood eating behaviors (25), and genetic and antenatal factors are important contributors to childhood eating behaviors (2). Feeding practices influence the eating environment through food provisions and parent–child interactions around feeding (4). Considering the impact of eating behaviors on the development of childhood OW/OB, research endeavors to identify adaptable feeding practices that encourage the formation of healthy eating behaviors during childhood (2). Evidence shows that maternal feeding practices may influence the development of childhood eating behaviors, which is associated with the risk of childhood OW/OB (26). Eating behaviors in infancy and young childhood are heritable traits; healthy eating behaviors is an important feeding strategy to combat childhood OW/OB (7). Eating behaviors influence OW/OB and disordered eating patterns, hence, it is imperative to identify modifiable feeding practices during infancy and young childhood that may promote the development of a healthy appetite and regulation of energy intake later in childhood and adulthood (2). Mothers often perceive their children as being attracted to food, e.g., higher score for the “enjoyment of food,” lower scores for the “satiety responsiveness,” “slowness in eating” and “food fussiness,” show that higher eating rates in children contribute to increase energy intake and are associated with higher BMI (27).

In contemporary temperament models, childhood temperament interacts with the parenting environment and contributes to early childhood development (4). Childhood temperament may have positive or negative health consequences depending on feeding practices (4). Temperamental avoidance of novelty appears to be strongly associated with negative responses to new foods after the emergence of wariness and self-locomotion (28). The relationship between negative childhood temperament and OW/OB has produced mixed results, as negative emotionality is related to both emotional overeating and undereating (29). Eating behaviors have been found to be related to temperament, which is distinct from individual differences referred to as “appetitive traits” (30). Higher negative levels of temperament are associated with increased food approach and higher food intake (15). Temperament interacts with the parenting environment, influencing childhood eating behaviors and OW/OB; and may evoke parental feeding decisions, influence childhood dysregulated eating and OW/OB (4). Therefore, when examining the predictors of childhood eating behaviors and OW/OB, it is important to consider the impact of temperament (4).

Pathways to childhood OW/OB include the environmental models, in which feeding practices are daily interactional activities dependent on the parenting environment (4). The existing body of research on the relationship between feeding practices and childhood eating behaviors primarily originates from studies conducted in developed western countries, with a specific emphasis on childhood OW/OB (17). Whereas an association between childhood temperament, feeding practices, childhood eating behaviors, and OW/OB may exist (4), with a significant implication in constructing the pathways from feeding practices to childhood OW/OB. Efforts to prevent childhood OW/OB have limited the consideration of maternal feeding practices through childhood eating behaviors and temperament. Sociocultural circumstances are critical in shaping maternal feeding practices, childhood eating behaviors, and OW/OB (31). Hence, understanding the local feeding context is imperative, considering the impact of ethnicity on childhood eating behaviors and preferences (31). The fast-tracked urbanization and lifestyle changes in China may disrupt for childhood access to food and feeding practices.

**Figure 1:**
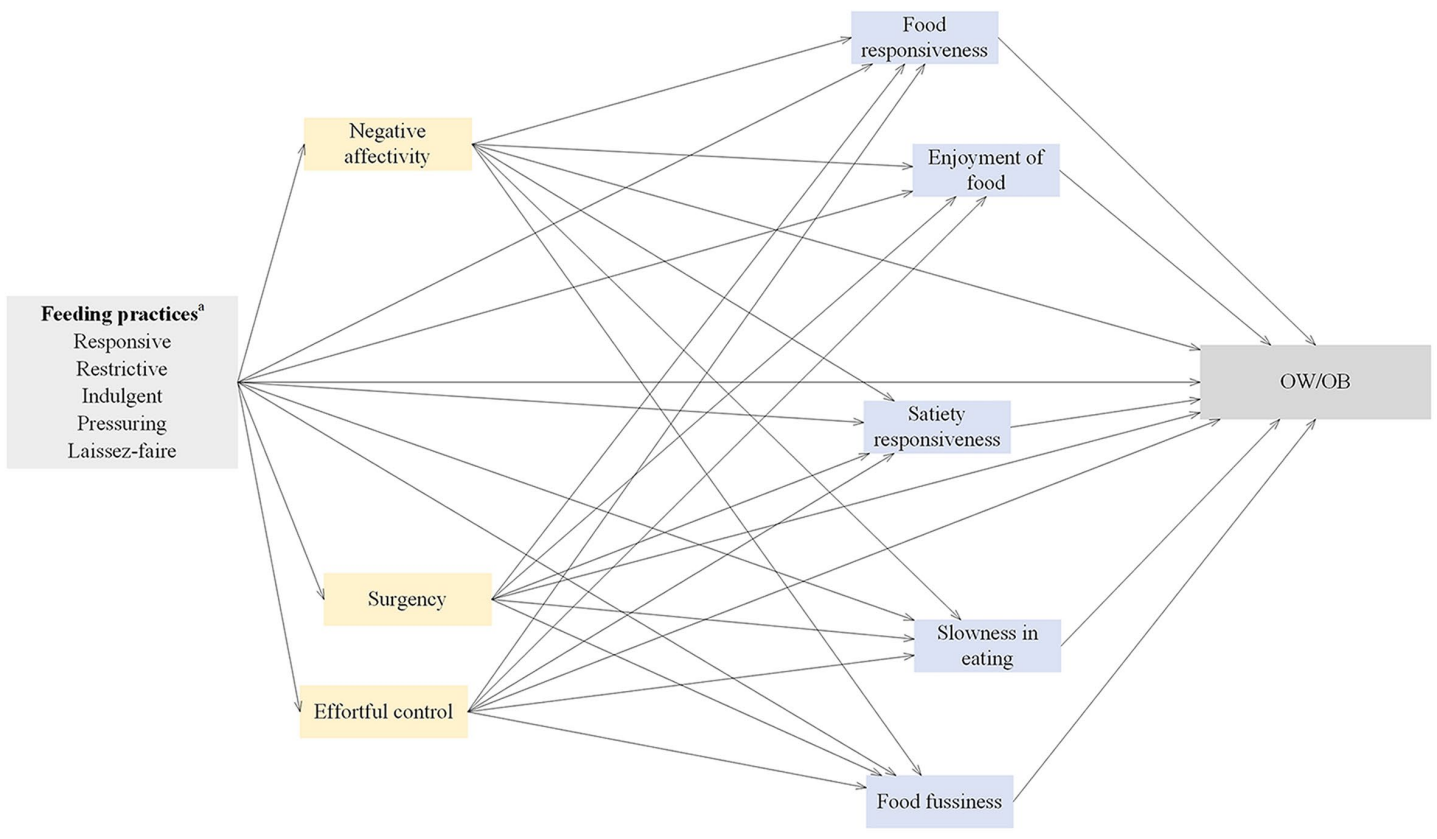
Conceptual framework and hypotheses. ^a^All feeding practices were examined in separate structural equation models. OW/OB, overweight and obesity.

To the best of our knowledge, to date, no study has explored the effect of feeding practices on childhood OW/OB to examine the mediators of temperament and eating behaviors in children aged 6–23 months (32). Developmental reactivity and regulation contribute to refining the conceptual framework of the associations between feeding practices, childhood temperament, eating behaviors, and OW/OB (33). Examining the impact of multiple feeding styles simultaneously on childhood OW/OB may be critical for exploring the mediators of childhood temperament and eating behaviors (34). Socioeconomic status has been found to predict an increase in emotional overeating and food responsiveness during childhood (35). Therefore, it is crucial to appropriately control for the impact of the differences in sociodemographic characteristics, which allows for a more accurate understanding of independent effects of feeding practices on childhood eating behaviors (2). This study provided a conceptual framework that aimed to explain the pathways from feeding practices to childhood OW/OB (33) with regard to childhood temperament and eating behaviors (Figure 1) as follows: (1) there may exist a direct effect from feeding practices to childhood OW/OB; (2) five styles of feeding practices may indirectly predict childhood OW/OB through the sequential mediation of three domains of temperament and five traits of eating behaviors.

## Methods

### Participants and procedure

This cross-sectional study was a snapshot taken at a point when vaccination was necessary for children, recruited mothers who were mainly responsible for child care (*n* = 486) from two hospitals (*n* = 234) and two healthcare centers (*n* = 252) in Xuzhou and Suqian, Jiangsu, China between April 5, 2020, and May 31, 2021. A purposive sampling method was used to recruit participants during vaccination. Inclusion criteria were as follows: mothers older than 18 years, with at least one child aged 6–23 months, born full-term with a birth weight of 2,500–4,000 g. Exclusion criteria were mothers with mental illness or children with health issues.

### Sample size

The sample size was calculated based on the prevalence of childhood OW and OB (15%), according to the calculation formula (36). The marginal error was within 3.2% with a 95% confidence level, *p* = 0.5; thus, the estimated maximum sample size was 479.

### Measures

#### Sociodemographic characteristics

The sociodemographic characteristics collected included: child age, sex (female, male), maternal age, education level (middle school or below, high school, college/university or above), employment status (unemployed, employed), area of residence (urban, rural) and annual household income (<100,000, 100,000–150,000, >150,000 RMB).

#### Maternal feeding practices

The Infant Feeding Style Questionnaire (IFSQ) is an 83-item measure, including 5 maternal feeding styles (37): responsive (12 items), restrictive (11 items), indulgent (32 items), pressuring (17 items), and laissez-faire (11 items). Each item was coded on a 5-point Likert scale ranging from 1 (*strongly disagree/never*) to 5 (*strongly agree/always*), each subscale was scored as the mean of responses, and higher scores indicated higher level of each feeding style. The IFSQ has been validated in Chinese mothers and the Cronbach’s alpha value was higher than 0.898 for each subscale (7).

#### Childhood temperament

The short form of Infant Behavior Questionnaire-Revised – (Revised IBQ-RSF) (11) with 91 items, and the Early Child Behavior Questionnaire (ECBQ-SF) (38) with 107 items were used to assess the childhood temperament of children aged between 6–15, and 16–24 months, respectively. The IBQ-RSF and ECBQ-SF included three dimensions: negative affectivity, surgency and effortful control, rated on a 7-point Likert scale ranging from 1 (*never*) to 7 (*always*). The negative affectivity consisted of four subscales assessing infant sadness, falling reactivity, fear, and distress to limitations (39). Surgency/Extraversion consisted of six subscales, assessing infant approach, high-intensity pleasure, vocal reactivity, smiling and laughter, activity level, and perceptual sensitivity (39). Orienting/Regulation consisted of four subscales of infants’ cuddliness/affiliation, duration of orientation, low-intensity pleasure, and soothability (39). Higher mean scores indicated a higher presence of temperamental traits. In this study, the Cronbach’s alpha values for negative affectivity, surgency, and effortful control in the IBQ-RSF were 0.94, 0.73 and 0.85 respectively; in the ECBQ-SF were 0.91, 0.82, and 0.87, respectively, which showed that IBQ-RSF was validated in Chinese children.

#### Childhood eating behaviors

The Child Eating Behavior Questionnaire for toddler (CEBQ-T) is a 26-item measure, including six subscales (16): food responsiveness (4 items), enjoyment of food (4 items), emotional overeating (3 items), satiety responsiveness (5 items), slowness in eating (4 items), and food fussiness (6 items). As mothers reported that their children did not engage in emotional overeating, this subscale was removed. Items were scored on a 5-point Likert scale ranging from 1 (*never*) to 5 (*always*), and higher mean scores indicated higher expression of eating behaviors. In this study, Cronbach’s alpha values for food responsiveness, enjoyment of food, satiety responsiveness, slowness in eating, and food fussiness were 0.76, 0.78, 0.86 0.88 and 0.90, respectively. A five-factor model’s confirmatory factor analysis (CFA) of indicated an acceptable model fit (Tables 1, 2).

#### Childhood OW/OB

The weight and recumbent length of children were measured following standard procedures, weight was measured while the children were wearing light clothes without shoes to the nearest 0.1 kg using a calibrated digital scale (Seca 354, China), and length was measured to the nearest 0.1 cm using a stadiometer (Seca 416, China) (40). The weight and recumbent length were measured in duplicate and recorded as the average values, and a third measurement was undertaken in case of a difference higher than 0.05 kg for weight or 1 cm for length. The average of the two closest values was used when three measurements were made. The age- and sex-specific BMI *z*-scores (BMIz) were calculated based on the World Health Organization (WHO) Child Growth Standards, and children were classified into overweight and obese (OW/OB; BMIz > +2) and non-OW/OB (BMIz ≤ +2) (41).

### Data analyses

Statistical analyses were performed using the STATA 15.0 software (Stata Corporation, College Station, TX, USA) and AMOS 23.0 software (IBM Corporation, Armonk, NY, USA). Data were double-checked for any errors, no missing values were reported in this study. Sociodemographic differences between hospitals and healthcare centers were examined using ANOVA and chi-square test. Pearson’s correlation analysis was conducted to evaluate the relationships among sociodemographic characteristics, feeding practices, temperament, eating behaviors, and OW/OB in childhood.

The structural equation modeling (SEM) was used with the maximum likelihood estimation to examine the direct and indirect pathways from five maternal feeding styles to childhood OW/OB through temperament and eating behaviors. Sociodemographic variables were included in the SEM as covariates if they were significantly associated with mediators (temperament and eating behaviors) or outcome variable (childhood OW/OB). Multiple goodness-of-fit indices were used to achieve the acceptable model fit, including the Comparative Fit Index (CFI) > 0.90, Adjusted Goodness-of-fit Index (AGFI) > 0.90, Goodness-of-fit Index (GFI) > 0.90, Root Mean Square Error of Approximation (RMSEA) < 0.08 and Standardized Root Mean Square Residual (SRMR) < 0.08 (42, 43). The bootstrap *resampling* procedures (*n* = 2000 samples) and a bias-corrected 95% confidence interval (BC 95% CI) were used to estimate the significant effects (44, 45). The total effects were calculated as the sum of the direct and indirect effects, mathematically expressed as follows: c = c′ + ab, where c = total effect, c′ = direct effect, and ab = indirect effect (46). The ratio was calculated as 100 × (indirect effect/total effect). A value of *p* < 0.05 indicated a statistically significant difference. According to the empirical estimates of the sample size for mediation models, the sample size of this study was 486 exceeding the required size for small-to-medium a and b paths with power = 0.80 (47).

A pilot study was conducted with a sample of 10 mothers to enhance the clarity and consistency of the questionnaires. The aim of the pilot study was to ensure a comprehensive understanding of the CEBQ-T. No amendments were necessary for the questionnaires and the data collected from the pilot study were integrated into the final sample for subsequent analysis.

### Ethics approval

Potential mothers were approached through word of mouth and flyers distributed during child vaccination visits. All mothers were asked to participate; if they agreed, they were provided with an information sheet detailing the study’s objectives, procedures, expected outcomes, benefits and risks. All mothers provided written informed consent, and were informed of the right to withdraw at any given time. All data were anonymized concerning data protection. Researchers were available to assist if necessary, e.g., by answering questions about this study, and assessing the completeness of the questionnaires. Mothers who completed the questionnaires received 30 RMB as compensation for their participation in the study. This study was conducted in accordance with the Declaration of Helsinki and approved by the Xuzhou Medical University Ethics Committee (ID number: XZ2018728).

## Results

### Sociodemographic characteristics

Sociodemographic differences between hospitals and healthcare centers are presented in Table 3. A total of 73 (15.02%) children were OW/OB; the age of children was 14.55 (*SD* = 5.14) months and 260 (53.50%) were males. The age of the mothers was 29.90 (*SD* = 3.63) years, 168 (34.57%) attended a high school, 264 (54.32%) were unemployed, 288 (59.26%) resided in urban areas, and 201 (41.36%) reported household income of less than 100,000 RMB per year. There was no statistically significant difference in sociodemographic comparison between hospitals and healthcare centers (Table 3).

**Table 1:** Standardized factor loadings for items of the CEBQ-T according to confirmatory factor analysis – 5 factors, 23 items^a^.

Factor	Item	Loading	Mean	SD
Food responsiveness	My child is always asking for food	0.632	3.24	1.21
	If allowed to, my child would eat too much	0.710	2.49	1.16
	Given the choice, my child would eat most of the time	0.597	2.48	1.27
	Even if my child is full up s/he finds room to eat his/her favorite food	0.715	2.70	1.31
Enjoyment of food	My child loves food	0.621	3.81	0.91
	My child is interested in food	0.858	3.82	0.94
	My child looks forward to mealtimes	0.561	2.80	1.10
	My child enjoys eating	0.720	3.66	0.91
Satiety responsiveness	My child has a big appetite^b^	0.696	3.01	0.97
	My child leaves food on his/her plate or in the jar at the end of a meal	0.756	2.98	0.98
	My child gets full before his/her meal is finished	0.799	2.96	0.93
	My child gets full up easily	0.738	3.07	0.92
	My child cannot eat a meal if s/he has had a snack just before	0.714	2.97	1.16
Slowness in eating	My child finishes his/her meal quickly^b^	0.789	2.72	1.16
	My child eats slowly	0.835	2.56	1.08
	My child takes more than 30 min to finish a meal	0.793	2.32	1.23
	My child eats more and more slowly during the course of a meal	0.822	2.42	1.12
Food fussiness	My child refuses new foods at first	0.701	2.01	0.99
	My child enjoys tasting new foods^b^	0.794	2.54	0.99
	My child enjoys a wide variety of foods^b^	0.794	2.42	1.10
	My child is difficult to please with meals	0.713	2.26	1.05
	My child is interested in tasting food s/he has not tasted before^b^	0.849	2.66	1.07
	My child decides that s/he does not like a food, even without tasting it	0.751	1.86	0.91

SD, standard deviation. ^a^The confirmatory factor analysis revealed that model fit was acceptable, with CFI = 0.957, AGFI = 0.903, GFI = 0.923, RMSEA = 0.044, and SRMR = 0.0454. ^b^Item is reverse coded.

**Table 2:** Factor-factor correlations of 5 eating behaviors (based on 23 items, CEBQ-T).

Factor		1	2	3	4	5
1	Food responsiveness	1.00				
2	Enjoyment of food	0.18**	1.00			
3	Satiety responsiveness	−0.28**	0.14*	1.00		
4	Slowness in eating	0.09	0.16**	0.11	1.00	
5	Food fussiness	−0.12*	−0.09	0.10	0.13*	1.00

*p < 0.05, **p < 0.01.

**Table 3:** Sociodemographic characteristics by hospitals and healthcare centers (*N* = 486).

Variables	Total	Healthcare center in Xuzhou (*n* = 208)	Hospital in Xuzhou (*n* = 161)	Healthcare center in Suqian (*n* = 44)	Hospital in Suqian (*n* = 73)	*F*/*χ*^2^	*p*
*Children*							
Age (months) *M* (SD)	14.55 (5.14)	14.49 (5.11)	14.48 (5.13)	14.18 (5.61)	15.12 (5.05)	0.396	0.756
*Sex n(%)*							
Male	260 (53.50)	105 (50.48)	89 (55.28)	22 (50.00)	44 (60.27)	2.530	0.470
Female	226 (46.50)	103 (49.52)	72 (44.72)	22 (50.00)	29 (39.73)		
*Weight groups^a^ n(%)*							
OW/OB	73 (15.02)	27 (12.98)	28 (17.39)	6 (13.64)	12 (16.44)	1.568	0.667
Non-OW/OB	413 (84.98)	181 (87.02)	133 (82.61)	38 (86.36)	61 (83.56)		
*Mothers*							
Age (years) *M* (SD)	29.90 (3.63)	30.00 (3.37)	29.97 (3.73)	29.89 (4.22)	29.45 (3.80)	0.440	0.724
*Education n (%)*							
Middle school or below	154 (31.69)	59 (28.37)	49 (30.43)	18 (40.91)	28 (38.36)	6.098	0.412
High school	168 (34.57)	75 (36.06)	53 (32.92)	16 (36.36)	24 (32.88)		
College/university or above	164 (33.74)	74 (35.58)	59 (36.65)	10 (22.73)	21 (28.77)		
*Employment status n (%)*							
Unemployed	264 (54.32)	113 (54.33)	84 (52.17)	24 (54.55)	43 (58.90)	0.918	0.821
Employed	222 (45.68)	95 (45.67)	77 (47.83)	20 (45.45)	30 (41.10)		
*Area of residence n (%)*							
Urban	288 (59.26)	131 (62.98)	94 (58.39)	22 (50.00)	41 (56.16)	3.096	0.377
Rural	198 (40.74)	77 (37.02)	67 (41.61)	22 (50.00)	32 (43.84)		
*Annual household income (RMB) n (%)*							
< 100,000	201 (41.36)	78 (37.5)	66 (40.99)	24 (54.55)	33 (45.21)	6.161	0.405
100,000–150,000	187 (38.48)	86 (41.35)	60 (37.27)	12 (27.27)	29 (39.73)		
> 150,000	98 (20.16)	44 (21.15)	35 (21.74)	8 (18.18)	11 (15.07)		

OW/OB, overweight and obesity; M (SD), mean (standard deviation). ^a^The age- and sex-specific body mass index z-score (BMIz) was calculated based on the World Health Organization Child Growth Standards, and children were classified into OW/OB (BMIz > +2) and non-OW/OB (BMIz ≤ +2) groups.

### Pearson’s correlation analyses

The results of the correlation analysis between sociodemographic characteristics, feeding practices, temperament, eating behaviors, and childhood OW/OB are listed in Table 4. Childhood OW/OB was positively correlated with restrictive feeding (*r* = 0.33, *p* < 0.05), indulgent feeding (*r* = 0.30, *p* < 0.05), food responsiveness (*r* = 0.27, *p* < 0.05), negative affectivity (*r* = 0.25, *p* < 0.05), and surgency (*r* = 0.13, *p* < 0.05); and negatively correlated with children sex (*r*  = − 0.28, *p*  <  0.05), maternal education (*r*  = −0.14, *p*  <  0.05), responsive feeding (*r*  = −0.23, *p*  <  0.05), pressuring feeding (*r*  = −0.23, *p*  <  0.05), effortful control (*r * = −0.34, *p*  <  0.05), satiety responsiveness (*r*  = −0.35, *p * <  0.05), slowness in eating (*r * = −0.17, *p*  <  0.05), and food fussiness (*r*  = −0.24, *p * <  0.05). Maternal education was positively correlated with effortful control (*r* = −0.14, *p* < 0.05). Children sex and maternal education were included as covariates in the SEM approach.

**Table 4:** Descriptive statistics and correlations of sociodemographic characteristics, feeding practices, temperament, eating behaviors, and OW/OB in childhood.

Variables		Mean	SD	1	2	3	4	5	6	7	8	9	10	11	12	13	14	15	16	17	18	19	20	21
1	Childhood OW/OB	-	-	1.00	0.27**	0.06	−0.35**	−0.17**	−0.24**	0.25**	0.13**	−0.34**	−0.23**	0.33**	0.30**	−0.23**	0.07	0.06	−0.28**	−0.07	−0.14**	−0.02	0.04	−0.01
*Childhood eating behaviors*																								
2	Food responsiveness	2.73	0.96	-	1.00	0.11*	−0.20**	−0.08	−0.09	0.22**	0.22**	−0.27**	−0.17**	0.14**	0.14**	−0.04	0.02	−0.01	−0.06	0.01	−0.08	−0.01	0.07	−0.05
3	Enjoyment of food	3.53	0.76	-	-	1.00	−0.08	0.13**	−0.06	0.11*	0.13**	−0.11*	0.08	0.10*	0.09*	−0.01	0.03	−0.04	0.01	−0.05	−0.03	−0.06	−0.07	0.04
4	Satiety responsiveness	3.00	0.76	-	-	-	1.00	0.13**	0.01	−0.06	−0.06	0.31**	0.20**	−0.18**	−0.05	0.07	−0.06	0.03	0.05	< 0.001	0.04	−0.01	−0.07	−0.01
5	Slowness in eating	2.50	0.85	-	-	-	-	1.00	0.12**	0.02	−0.06	0.06	0.02	−0.07	−0.09	0.20**	−0.03	−0.01	< 0.001	0.02	−0.03	−0.07	−0.02	0.06
6	Food fussiness	2.29	0.67	-	-	-	-	-	1.00	−0.03	−0.13**	0.08	0.05	−0.05	−0.10*	0.23**	−0.05	0.02	0.04	0.02	0.07	0.06	−0.02	0.07
*Childhood temperament*																								
7	Negative affectivity	3.51	1.27	-	-	-	-	-	-	1.00	0.07	−0.13**	−0.21**	0.06	0.06	−0.07	0.04	< 0.001	−0.06	0.03	−0.02	0.02	−0.01	0.06
8	Surgency	4.97	0.83	-	-	-	-	-	-	-	1.00	−0.15**	−0.07	0.07	0.18**	−0.07	−0.03	−0.07	−0.03	−0.05	< 0.001	−0.03	0.02	−0.01
9	Effortful control	4.72	0.86	-	-	-	-	-	-	-	-	1.00	0.19**	−0.19**	−0.05	0.06	−0.05	0.04	0.05	0.05	0.14**	0.05	−0.05	0.04
*Feeding practices*																								
10	Responsive	4.04	0.67	-	-	-	-	-	-	-	-	-	1.00	−0.09*	−0.09*	0.08	0.02	0.03	0.00	0.04	0.06	0.03	0.03	−0.04
11	Restrictive	3.66	0.78	-	-	-	-	-	-	-	-	-	-	1.00	0.10*	−0.09*	0.14**	0.05	−0.07	0.02	−0.06	0.00	0.01	0.04
12	Indulgent	1.79	0.69	-	-	-	-	-	-	-	-	-	-	-	1.00	−0.12**	0.01	0.03	−0.08	−0.06	−0.05	0.01	0.04	0.09*
13	Pressuring	2.89	0.77	-	-	-	-	-	-	-	-	-	-	-	-	1.00	−0.01	−0.05	0.07	−0.01	0.07	−0.01	−0.06	0.03
14	Laissez-faire	2.03	0.80	-	-	-	-	-	-	-	-	-	-	-	-	-	1.00	0.00	0.01	0.01	−0.04	−0.01	−0.01	−0.06
*Children*																								
15	Age	-	-	-	-	-	-	-	-	-	-	-	-	-	-	-	-	1.00	−0.09*	0.08	−0.06	0.03	0.06	0.07
16	Sex	-	-	-	-	-	-	-	-	-	-	-	-	-	-	-	-	-	1.00	0.01	0.11*	0.04	< 0.001	0.04
*Mothers*																								
17	Age	-	-	-	-	-	-	-	-	-	-	-	-	-	-	-	-	-	-	1.00	−0.02	−0.02	−0.06	−0.05
18	Education	-	-	-	-	-	-	-	-	-	-	-	-	-	-	-	-	-	-	-	1.00	−0.02	−0.02	0.06
19	Employment status	-	-	-	-	-	-	-	-	-	-	-	-	-	-	-	-	-	-	-	-	1.00	0.04	0.05
20	Area of residence	-	-	-	-	-	-	-	-	-	-	-	-	-	-	-	-	-	-	-	-	-	1.00	−0.04
21	Annual household income	-	-	-	-	-	-	-	-	-	-	-	-	-	-	-	-	-	-	-	-	-	-	1.00

Gender: 0 = boy, 1 = girl; Education: 0 = Middle school or below, 1 = High school, 2 = College/university or above; Employment status: 0 = unemployed, 1 = employed; Area of residence: 0 = urban, 1 = rural; Annual household income: 0 = < 100,000RMB, 1 = 100,000–150,000RMB, > 150,000RMB. OW/OB, overweight and obesity; SD, standard deviation. ^*^p < 0.05, ^**^p < 0.01.

### SEM analyses

#### Pathways from responsive feeding to childhood OW/OB

**Figure 2:**
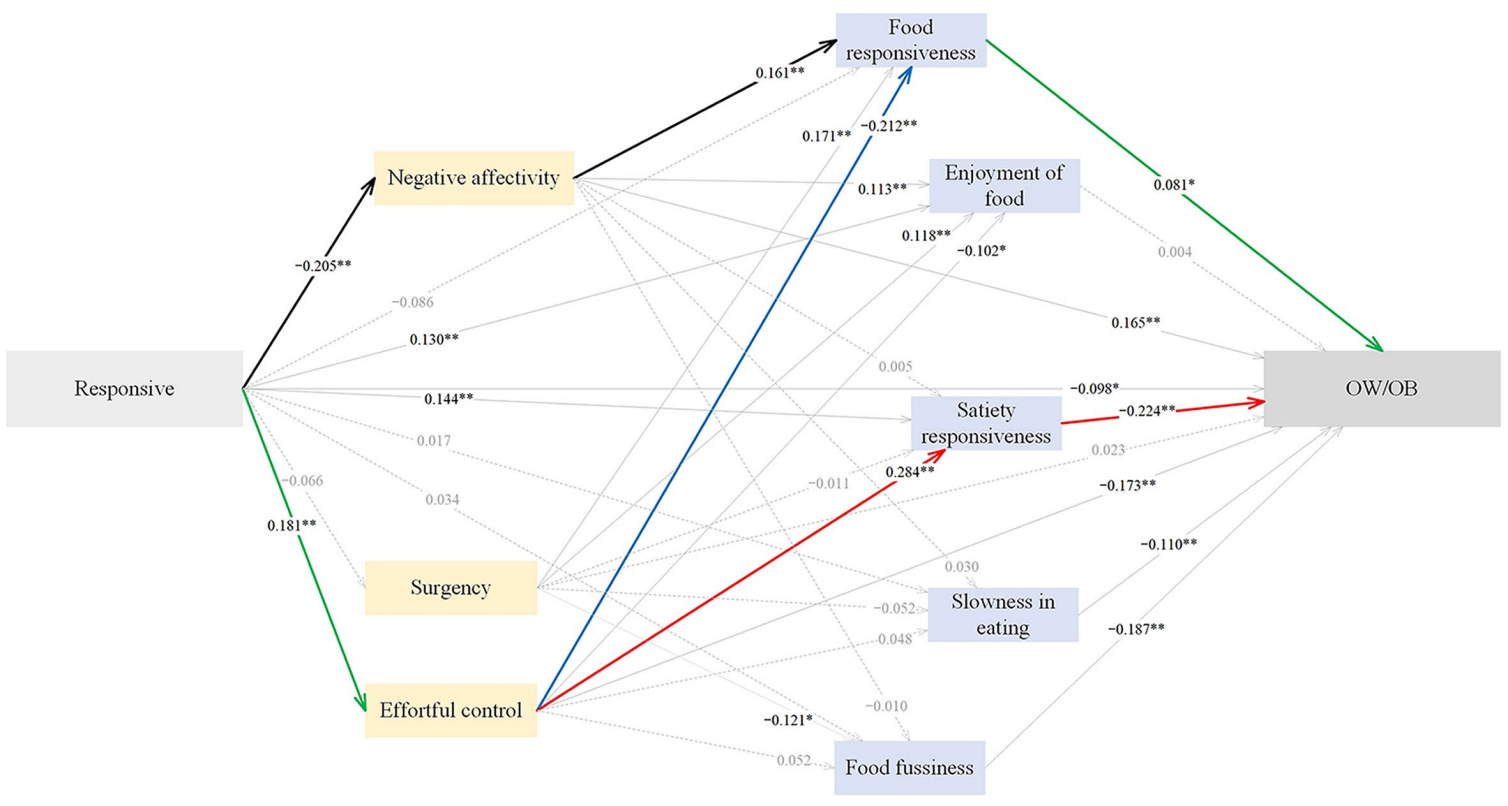
The standardized direct effects of responsive feeding on OW/OB via temperament and eating behaviors in childhood. For simplicity the covariates (children sex and maternal education) and measurement error terms are not shown. Dotted lines depict non-significant direct effect. Solid lines denote significant direct effect, and the bold black, green, blue and red solid lines represent sequential mediation effects. Model fit: CFI = 0.914, AGFI = 0.943, GFI = 0.977, RMSEA = 0.050, SRMR = 0.0455. OW/OB, overweight and obesity. *p < 0.05, **p < 0.01.

The standardized path estimates of the pathways from responsive feeding to childhood OW/OB are shown in Figure 2 and Table 5. Responsive feeding exerted significant direct (*β* = −0.098), indirect (*β* = −0.136), and total (*β* = −0.234) effects on childhood OW/OB. 41.88% of the total effect was direct, and 58.12% was indirect. Responsive feeding negatively predicted childhood OW/OB through lower negative affectivity (*β* = −0.034), higher satiety responsiveness (*β* = −0.032), and higher effortful control (*β* = −0.031), explaining 14.53, 13.68, and 13.25% of the total effect, respectively. Responsive feeding negatively predicted childhood OW/OB through the sequential mediation of higher effortful control and higher satiety responsiveness (*β* = −0.012), accounting for 5.13% of the total effect; the sequential mediation of higher effortful control and lower food responsiveness (*β* = −0.003), explaining 1.28% of the total effect; the sequential mediation of lower negative affectivity and lower food responsiveness (*β* = −0.003), accounting for 1.28% of the total effect. The SEM approach indicated an acceptable model fit, with CFI = 0.914, AGFI = 0.943, GFI = 0.977, RMSEA = 0.050 and SRMR = 0.046.

**Table 5:** Standardized indirect and total effects of responsive feeding on OW/OB via temperament and eating behaviors in childhood^a^.

Effects	Paths	*β*	*SE*	*p*	BC 95% CI	Ratio (*100) Specific indirect effect to total effect^b^ (%)
Indirect effects	**Responsive → Negative affectivity → OW/OB**	**−0.034**	**0.012**	**0.001**	**−0.061 to −0.014**	**14.530**
	Responsive → Surgency → OW/OB	−0.002	0.003	0.311	−0.013 to 0.003	-
	**Responsive → Effortful control → OW/OB**	**−0.031**	**0.012**	**< 0.001**	**−0.063 to −0.012**	**13.248**
	Responsive → Food responsiveness → OW/OB	−0.007	0.005	0.050	−0.022 to <0.001	-
	Responsive → Enjoyment of food → OW/OB	0.001	0.005	0.872	−0.010 to 0.012	-
	**Responsive → Satiety responsiveness → OW/OB**	**−0.032**	**0.012**	**0.001**	**−0.060 to −0.012**	**13.675**
	Responsive → Slowness in eating → OW/OB	−0.002	0.005	0.619	−0.014 to 0.007	-
	Responsive → Food fussiness → OW/OB	−0.006	0.008	0.453	−0.024 to 0.010	-
	**Responsive → Negative affectivity → Food responsiveness → OW/OB**	**−0.003**	**0.002**	**0.014**	**−0.007 to −0.001**	**1.282**
	Responsive → Negative affectivity → Enjoyment of food → OW/OB	< 0.001	0.001	0.844	−0.002 to 0.002	-
	Responsive → Negative affectivity → Satiety responsiveness → OW/OB	< 0.001	0.002	0.900	−0.004 to 0.004	-
	Responsive → Negative affectivity → Slowness in eating → OW/OB	0.001	0.001	0.379	−0.001 to 0.003	-
	Responsive → Negative affectivity → Food fussiness → OW/OB	< 0.001	0.002	0.849	−0.004 to 0.004	-
	Responsive → Surgency → Food responsiveness → OW/OB	−0.001	0.001	0.082	−0.004 to <0.001	-
	Responsive → Surgency → Enjoyment of food → OW/OB	< 0.001	< 0.001	0.699	−0.001 to 0.001	-
	Responsive → Surgency → Satiety responsiveness → OW/OB	< 0.001	0.001	0.621	−0.003 to 0.001	-
	Responsive → Surgency → Slowness in eating → OW/OB	< 0.001	0.001	0.170	−0.003 to <0.001	-
	Responsive → Surgency → Food fussiness → OW/OB	−0.001	0.001	0.103	−0.006 to <0.001	-
	**Responsive → Effortful control → Food responsiveness → OW/OB**	**−0.003**	**0.002**	**0.017**	**−0.009 to −0.001**	**1.282**
	Responsive → Effortful control → Enjoyment of food → OW/OB	< 0.001	0.001	0.822	−0.002 to 0.001	-
	**Responsive → Effortful control → Satiety responsiveness → OW/OB**	**−0.012**	**0.004**	**< 0.001**	**−0.022 to −0.005**	**5.128**
	Responsive → Effortful control → Slowness in eating → OW/OB	−0.001	0.001	0.173	−0.004 to 0.001	-
	Responsive → Effortful control → Food fussiness → OW/OB	−0.002	0.002	0.151	−0.006 to 0.001	-
Total effect	**Responsive → OW/OB**	**−0.234**	**0.044**	**0.001**	**−0.324 to −0.149**	**100**

OW/OB, overweight and obesity; SE, standard error; BC 95% CI, bias-corrected 95% confidence interval. ^a^When *p* < 0.05, the path, *β*, SE, *p*, BC 95% CI and ratio (*100) Specific Indirect Effect to Total Effect are bolded. ^b^Ratio was calculated as 100 × (indirect effect (*β*)/total effect), where the total effect was the sum of the direct and indirect effects.

#### Pathways from restrictive feeding to childhood OW/OB

**Figure 3:**
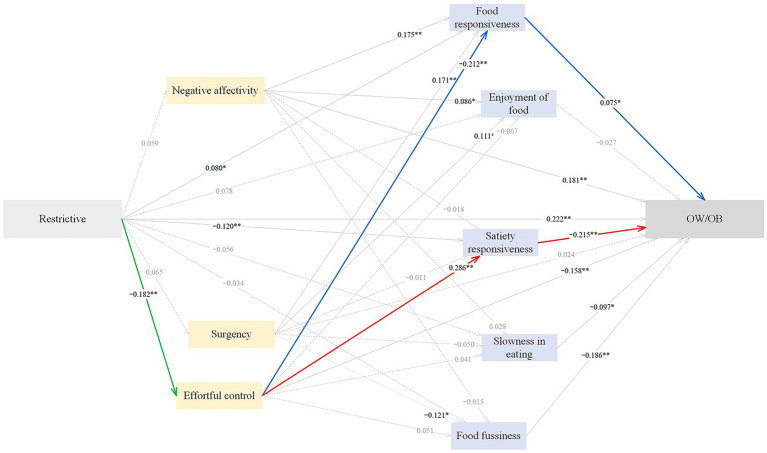
The standardized direct effects of restrictive feeding on OW/OB via temperament and eating behaviors in childhood. For simplicity the covariates (children sex and maternal education) and measurement error terms are not shown. Dotted lines depict non-significant direct effect. Solid lines denote significant direct effect, and the bold green, blue and red solid lines represent sequential mediation effects. Model fit: CFI = 0.913, AGFI = 0.942, GFI = 0.977, RMSEA = 0.050, SRMR = 0.0470. OW/OB, overweight and obesity. *p < 0.05, **p < 0.01.

The standardized path estimates of the pathways from restrictive feeding to childhood OW/OB are shown in Figure 3 and Table 6. Restrictive feeding had significant direct (*β* = 0.222), indirect (*β* = 0.102) and total (*β* = 0.324) effects on childhood OW/OB; 68.52% of the total effect was direct, and 31.48% was indirect. Restrictive feeding positively predicted childhood OW/OB through lower effortful control (*β* = 0.029), lower satiety responsiveness (*β* = 0.026), and higher food responsiveness (*β* = 0.006), accounting for 8.95, 8.03 and 1.85% of the total effect; the sequential mediation of lower effortful control and lower satiety responsiveness (*β* = 0.011), accounting for 3.40% of the total effect; the sequential mediation of lower effortful control and higher food responsiveness (*β* = 0.003), explaining 0.93% of the total effect. The SEM approach indicated an acceptable model fit, with CFI = 0.913, AGFI = 0.942, GFI = 0.977, RMSEA = 0.050, and SRMR = 0.047.

**Table 6:** Standardized indirect and total effects of restrictive feeding on OW/OB via temperament and eating behaviors in childhood^a^.

Effects	Paths	*β*	*SE*	*p*	BC 95% CI	Ratio (*100) Specific indirect effect to total effect^b^ (%)
Indirect effects	Restrictive → Negative affectivity → OW/OB	0.011	0.009	0.143	−0.004 to 0.031	-
	Restrictive → Surgency → OW/OB	0.002	0.003	0.286	−0.002 to 0.014	-
	**Restrictive → Effortful control → OW/OB**	**0.029**	**0.012**	**0.001**	**0.012 to 0.059**	**8.951**
	**Restrictive → Food responsiveness → OW/OB**	**0.006**	**0.004**	**0.036**	**< 0.001 to 0.019**	**1.852**
	Restrictive → Enjoyment of food → OW/OB	−0.002	0.004	0.305	−0.013 to 0.003	-
	**Restrictive → Satiety responsiveness → OW/OB**	**0.026**	**0.011**	**0.002**	**0.008 to 0.052**	**8.025**
	Restrictive → Slowness in eating → OW/OB	0.005	0.005	0.149	−0.002 to 0.019	-
	Restrictive → Food fussiness → OW/OB	0.006	0.009	0.427	−0.010 to 0.026	-
	Restrictive → Negative affectivity → Food responsiveness → OW/OB	0.001	0.001	0.075	< 0.001 to 0.004	-
	Restrictive → Negative affectivity → Enjoyment of food → OW/OB	< 0.001	< 0.001	0.227	−0.001 to <0.001	-
	Restrictive → Negative affectivity → Satiety responsiveness → OW/OB	< 0.001	0.001	0.430	−0.001 to 0.003	-
	Restrictive → Negative affectivity → Slowness in eating → OW/OB	< 0.001	< 0.001	0.262	−0.002 to <0.001	-
	Restrictive → Negative affectivity → Food fussiness → OW/OB	< 0.001	0.001	0.515	−0.001 to 0.002	-
	Restrictive → Surgency → Food responsiveness → OW/OB	0.001	0.001	0.060	< 0.001 to 0.004	-
	Restrictive → Surgency → Enjoyment of food → OW/OB	< 0.001	< 0.001	0.241	−0.002 to <0.001	-
	Restrictive → Surgency → Satiety responsiveness → OW/OB	< 0.001	0.001	0.620	−0.001 to 0.003	-
	Restrictive → Surgency → Slowness in eating → OW/OB	< 0.001	< 0.001	0.146	< 0.001 to 0.002	-
	Restrictive → Surgency → Food fussiness → OW/OB	0.001	0.001	0.083	< 0.001 to 0.006	-
	**Restrictive → Effortful control → Food responsiveness → OW/OB**	**0.003**	**0.002**	**0.027**	**< 0.001 to 0.009**	**0.926**
	Restrictive → Effortful control → Enjoyment of food → OW/OB	< 0.001	0.001	0.289	−0.002 to <0.001	-
	**Restrictive → Effortful control → Satiety responsiveness → OW/OB**	**0.011**	**0.004**	**0.001**	**0.005 to 0.020**	**3.395**
	Restrictive → Effortful control → Slowness in eating → OW/OB	0.001	0.001	0.209	−0.001 to 0.004	-
	Restrictive → Effortful control → Food fussiness → OW/OB	0.002	0.002	0.187	−0.001 to 0.006	-
Total effect	**Restrictive → OW/OB**	**0.324**	**0.039**	**0.001**	**0.244 to 0.396**	**100**

OW/OB, overweight and obesity; SE, standard error; BC 95% CI, bias-corrected 95% confidence interval. ^a^When *p* < 0.05, the path, *β*, SE, *p*, BC 95% CI and ratio (*100) Specific Indirect Effect to Total Effect are bolded. ^b^Ratio was calculated as 100 × (indirect effect (*β*)/total effect), where the total effect was the sum of the direct and indirect effects.

#### Pathways from indulgent feeding to childhood OW/OB

**Figure 4:**
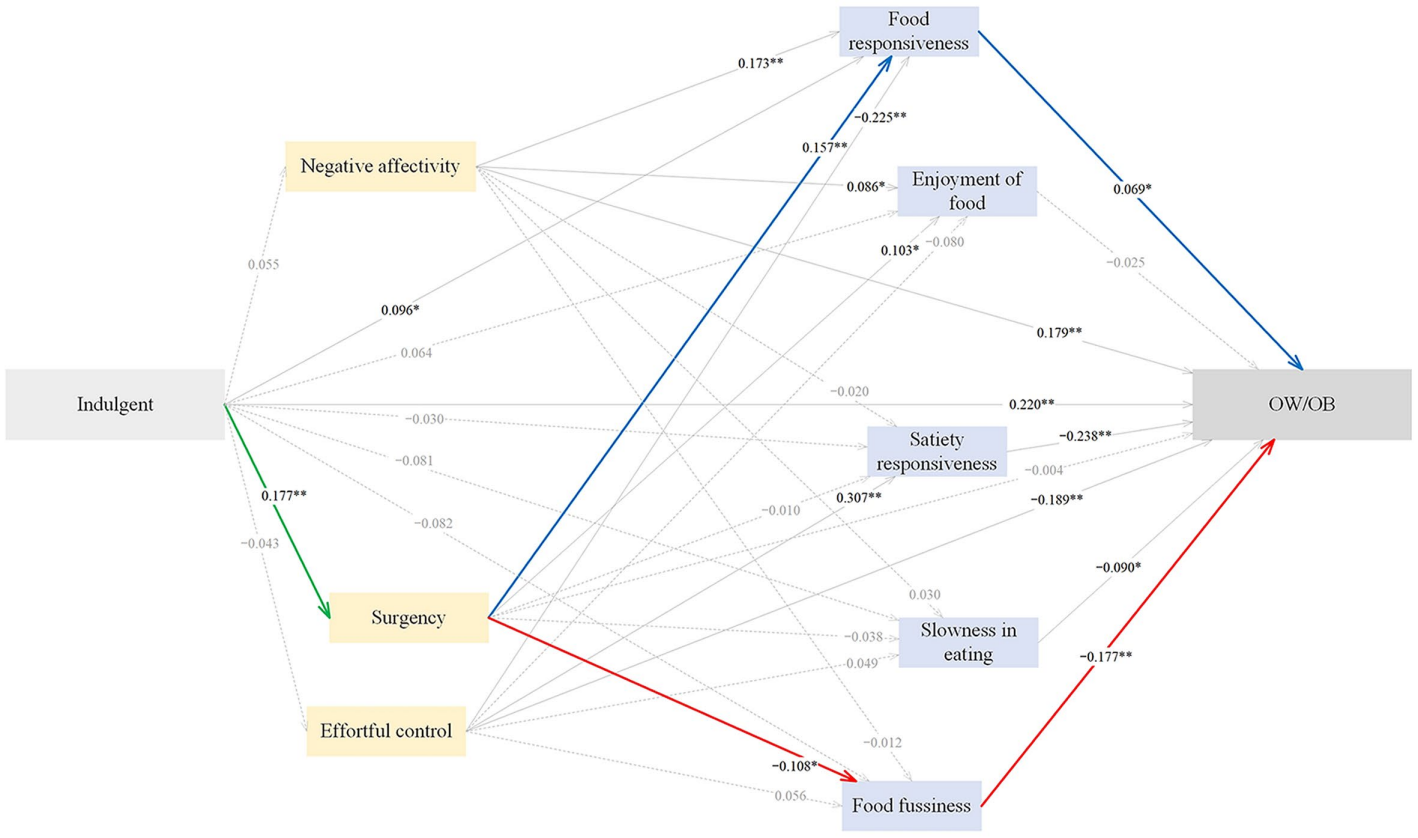
The standardized direct effects of indulgent feeding on OW/OB via temperament and eating behaviors in childhood. For simplicity the covariates (children sex and maternal education) and measurement error terms are not shown. Dotted lines depict non-significant direct effect. Solid lines denote significant direct effect, and the bold green, blue and red solid lines represent sequential mediation effects. Model fit: CFI = 0.908, AGFI = 0.940, GFI = 0.976, RMSEA = 0.052, SRMR = 0.0478. OW/OB, overweight and obesity. *p < 0.05, **p < 0.01.

The standardized path estimates of the pathways from pressuring feeding to childhood OW/OB are shown in Figure 4 and Table 7. Indulgent feeding had significant direct (*β* = 0.220), indirect (*β* = 0.063) and total (*β* = 0.283) effects on childhood OW/OB. 54.72% of the total effect was direct, and 22.26% was indirect. Indulgent feeding positively predicted childhood OW/OB through higher food fussiness (*β* = 0.007), accounting for 2.47% of the total effect; the sequential mediation of higher surgency and lower food fussiness (*β* = 0.003), explaining 1.06% of the total effect; the sequential mediation of higher surgency and higher food responsiveness (*β* = 0.002), accounting for 0.71% of the total effect. The SEM approach indicated an acceptable model fit, with CFI = 0.908, AGFI = 0.940, GFI = 0.976, RMSEA = 0.052, and SRMR = 0.048.

**Table 7:** Standardized indirect and total effects of indulgent feeding on OW/OB via temperament and eating behaviors in childhood^a^.

Effects	Paths	*β*	*SE*	*p*	BC 95% CI	Ratio (*100) Specific indirect effect to total effect^b^ (%)
Indirect effects	Indulgent → Negative affectivity → OW/OB	0.010	0.009	0.203	−0.007 to 0.031	-
	Indulgent → Surgency → OW/OB	−0.001	0.007	0.958	−0.016 to 0.013	-
	Indulgent → Effortful control → OW/OB	0.008	0.010	0.289	−0.008 to 0.033	-
	**Indulgent → Food responsiveness → OW/OB**	**0.007**	**0.005**	**0.048**	**< 0.001 to 0.021**	**2.473**
	Indulgent → Enjoyment of food → OW/OB	−0.002	0.003	0.292	−0.013 to 0.002	-
	Indulgent → Satiety responsiveness → OW/OB	0.007	0.012	0.511	−0.015 to 0.031	-
	Indulgent → Slowness in eating → OW/OB	0.007	0.005	0.066	< 0.001 to 0.021	-
	Indulgent → Food fussiness → OW/OB	0.015	0.009	0.077	−0.001 to 0.036	-
	Indulgent → Negative affectivity → Food responsiveness → OW/OB	0.001	0.001	0.120	< 0.001 to 0.003	-
	Indulgent → Negative affectivity → Enjoyment of food → OW/OB	< 0.001	< 0.001	0.247	−0.001 to <0.001	-
	Indulgent → Negative affectivity → Satiety responsiveness → OW/OB	< 0.001	0.001	0.382	−0.001 to 0.003	-
	Indulgent → Negative affectivity → Slowness in eating → OW/OB	< 0.001	< 0.001	0.254	−0.002 to <0.001	-
	Indulgent → Negative affectivity → Food fussiness → OW/OB	< 0.001	0.001	0.574	−0.001 to 0.002	-
	**Indulgent → Surgency → Food responsiveness → OW/OB**	**0.002**	**0.001**	**0.028**	**< 0.001 to 0.006**	**0.707**
	Indulgent → Surgency → Enjoyment of food → OW/OB	< 0.001	0.001	0.345	−0.003 to 0.001	-
	Indulgent → Surgency → Satiety responsiveness → OW/OB	< 0.001	0.002	0.807	−0.004 to 0.005	-
	Indulgent → Surgency → Slowness in eating → OW/OB	0.001	0.001	0.285	−0.001 to 0.004	-
	**Indulgent → Surgency → Food fussiness → OW/OB**	**0.003**	**0.002**	**0.027**	**< 0.001 to 0.009**	**1.060**
	Indulgent → Effortful control → Food responsiveness → OW/OB	0.001	0.001	0.188	< 0.001 to 0.004	-
	Indulgent → Effortful control → Enjoyment of food → OW/OB	< 0.001	< 0.001	0.260	−0.001 to <0.001	-
	Indulgent → Effortful control → Satiety responsiveness → OW/OB	0.003	0.004	0.308	−0.003 to 0.012	-
	Indulgent → Effortful control → Slowness in eating → OW/OB	< 0.001	< 0.001	0.209	< 0.001 to 0.002	-
	Indulgent → Effortful control → Food fussiness → OW/OB	< 0.001	0.001	0.214	< 0.001 to 0.003	-
Total effect	**Indulgent → OW/OB**	**0.283**	**0.043**	**0.001**	**0.194 to 0.367**	**100**

OW/OB, overweight and obesity; SE, standard error; BC 95% CI, bias-corrected 95% confidence interval. ^a^When *p* < 0.05, the path, *β*, SE, *p*, BC 95% CI and ratio (*100) Specific Indirect Effect to Total Effect are bolded. ^b^Ratio was calculated as 100 × (indirect effect (*β*)/total effect), where the total effect was the sum of the direct and indirect effects.

#### Pathways from pressuring feeding to childhood OW/OB

**Figure 5:**
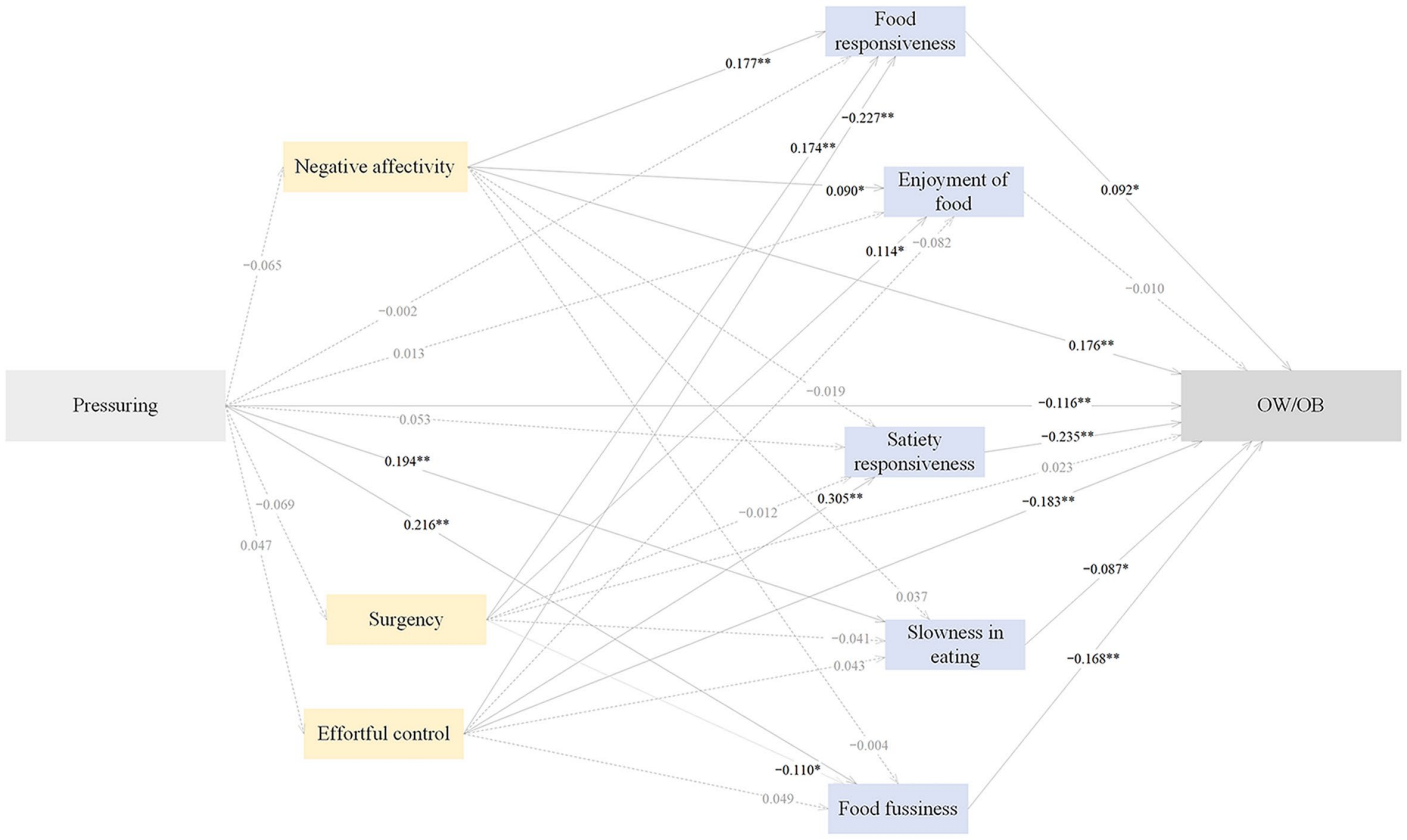
The standardized direct effects of pressuring feeding on OW/OB via temperament and eating behaviors in childhood. Note. For simplicity the covariates (children sex and maternal education) and measurement error terms are not shown. Dotted lines depict non-significant direct effect. Solid lines denote significant direct effect. Model fit: CFI = 0.909, AGFI = 0.941, GFI = 0.976, RMSEA = 0.051, SRMR = 0.0476. OW/OB, overweight and obesity. *p < 0.05, **p < 0.01.

The standardized path estimates of the pathways from pressuring feeding to childhood OW/OB through temperament and eating behaviors are shown in Figure 5 and Table 8. Pressuring feeding had significant direct (*β* = −0.116), indirect (*β* = −0.096) and total (*β* = −0.212) effects on childhood OW/OB. 54.72% of the total effect was direct, and 45.28% was indirect. Pressuring feeding negatively predicted childhood OW/OB through higher food fussiness (*β* = −0.036) and higher slowness in eating (*β* = −0.017), accounting for 16.98 and 8.02% of the total effect, respectively. The SEM approach indicated an acceptable model fit, with CFI = 0.909, AGFI = 0.941, GFI = 0.976, RMSEA = 0.051, and SRMR = 0.048.

**Table 8:** Standardized indirect and total effects of pressuring feeding on OW/OB via temperament and eating behaviors in childhood^a^.

Effects	Paths	*β*	*SE*	*P*	BC 95% CI	Ratio (*100) Specific indirect effect to total effect^b^ (%)
Indirect effects	Pressuring → Negative affectivity → OW/OB	−0.011	0.009	0.108	−0.033 to 0.003	-
	Pressuring → Surgency → OW/OB	−0.002	0.004	0.360	−0.014 to 0.003	-
	Pressuring → Effortful control → OW/OB	−0.009	0.009	0.239	−0.031 to 0.007	-
	Pressuring → Food responsiveness → OW/OB	< 0.001	0.004	0.916	−0.009 to 0.008	-
	Pressuring → Enjoyment of food → OW/OB	< 0.001	0.002	0.703	−0.005 to 0.002	-
	Pressuring → Satiety responsiveness → OW/OB	−0.013	0.011	0.181	−0.037 to 0.006	-
	**Pressuring → Slowness in eating → OW/OB**	**−0.017**	**0.009**	**0.029**	**−0.037 to −0.003**	**8.019**
	**Pressuring → Food fussiness → OW/OB**	**−0.036**	**0.012**	**0.001**	**−0.064 to −0.017**	**16.981**
	Pressuring → Negative affectivity → Food responsiveness → OW/OB	−0.001	0.001	0.057	−0.004 to <0.001	-
	Pressuring → Negative affectivity → Enjoyment of food → OW/OB	< 0.001	< 0.001	0.488	< 0.001 to 0.001	-
	Pressuring → Negative affectivity → Satiety responsiveness → OW/OB	< 0.001	0.001	0.448	−0.003 to 0.001	-
	Pressuring → Negative affectivity → Slowness in eating → OW/OB	< 0.001	< 0.001	0.185	< 0.001 to 0.002	-
	Pressuring → Negative affectivity → Food fussiness → OW/OB	< 0.001	0.001	0.803	−0.002 to 0.001	-
	Pressuring → Surgency → Food responsiveness → OW/OB	−0.001	0.001	0.069	−0.004 to <0.001	-
	Pressuring → Surgency → Enjoyment of food → OW/OB	< 0.001	< 0.001	0.516	< 0.001 to 0.001	-
	Pressuring → Surgency → Satiety responsiveness → OW/OB	< 0.001	0.001	0.585	−0.003 to 0.001	-
	Pressuring → Surgency → Slowness in eating → OW/OB	< 0.001	< 0.001	0.187	−0.003 to <0.001	-
	Pressuring → Surgency → Food fussiness → OW/OB	−0.001	0.001	0.082	−0.006 to <0.001	-
	Pressuring → Effortful control → Food responsiveness → OW/OB	−0.001	0.001	0.196	−0.005 to 0.001	-
	Pressuring → Effortful control → Enjoyment of food → OW/OB	< 0.001	< 0.001	0.440	< 0.001 to 0.001	-
	Pressuring → Effortful control → Satiety responsiveness → OW/OB	−0.003	0.003	0.265	−0.011 to 0.003	-
	Pressuring → Effortful control → Slowness in eating → OW/OB	< 0.001	< 0.001	0.201	−0.002 to <0.001	-
	Pressuring → Effortful control → Food fussiness → OW/OB	< 0.001	0.001	0.244	−0.003 to <0.001	-
Total effect	**Pressuring → OW/OB**	**−0.212**	**0.044**	**0.001**	**−0.298 to −0.126**	**100**

OW/OB, overweight and obesity; SE, standard error; BC 95% CI, bias-corrected 95% confidence interval. ^a^When *p* < 0.05, the path, *β*, SE, *p*, BC 95% CI and ratio (*100) Specific Indirect Effect to Total Effect are bolded. ^b^Ratio was calculated as 100 × (indirect effect (*β*)/total effect), where the total effect was the sum of the direct and indirect effects.

#### Pathways from laissez-faire feeding to childhood OW/OB

**Figure 6:**
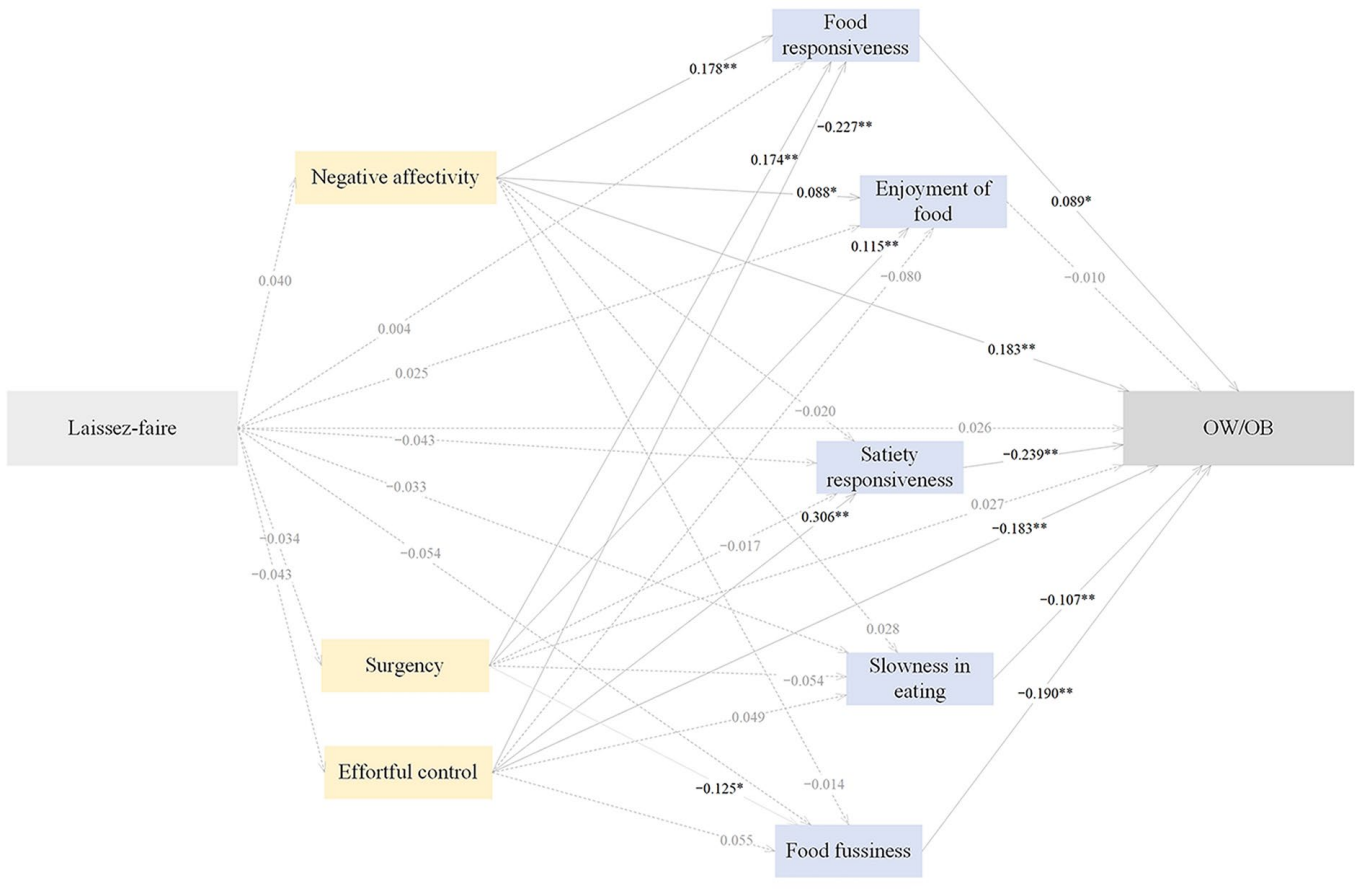
The standardized direct effects of laissez-faire feeding on OW/OB via temperament and eating behaviors in childhood. For simplicity the covariates (children sex and maternal education) and measurement error terms are not shown. Dotted lines depict non-significant direct effect. Solid lines denote significant direct effect. Model fit: CFI = 0.892, AGFI = 0.940, GFI = 0.976, RMSEA = 0.052, SRMR = 0.0483. OW/OB, overweight and obesity. *p < 0.05, **p < 0.01.

The pathways from laissez-faire feeding to childhood OW/OB through temperament and eating behaviors were non-significant (*p* > 0.05) (Figure 6; Table 9).

**Table 9:** Standardized indirect and total effects of laissez-faire feeding on OW/OB via temperament and eating behaviors in childhood^a^.

Effects	Paths	*β*	*SE*	*p*	BC 95% CI	Ratio (*100) Specific indirect effect to total effect^b^ (%)
Indirect effects	Laissez-faire → Negative affectivity → OW/OB	0.007	0.009	0.326	−0.009 to 0.028	-
	Laissez-faire → Surgency → OW/OB	−0.001	0.003	0.349	−0.011 to 0.002	-
	Laissez-faire → Effortful control → OW/OB	0.008	0.009	0.273	−0.007 to 0.033	-
	Laissez-faire → Food responsiveness → OW/OB	< 0.001	0.004	0.850	−0.008 to 0.009	-
	Laissez-faire → Enjoyment of food → OW/OB	< 0.001	0.002	0.636	−0.008 to 0.003	-
	Laissez-faire → Satiety responsiveness → OW/OB	0.010	0.011	0.311	−0.010 to 0.033	-
	Laissez-faire → Slowness in eating → OW/OB	0.004	0.005	0.388	−0.005 to 0.017	-
	Laissez-faire → Negative affectivity → Food responsiveness → OW/OB	0.001	0.001	0.198	< 0.001 to 0.003	-
	Laissez-faire → Negative affectivity → Enjoyment of food → OW/OB	< 0.001	< 0.001	0.458	−0.001 to <0.001	-
	Laissez-faire → Negative affectivity → Satiety responsiveness → OW/OB	< 0.001	0.001	0.456	−0.001 to 0.003	-
	Laissez-faire → Negative affectivity → Slowness in eating → OW/OB	< 0.001	< 0.001	0.298	−0.002 to <0.001	-
	Laissez-faire → Surgency → Food responsiveness → OW/OB	−0.001	0.001	0.327	−0.003 to 0.001	-
	Laissez-faire → Surgency → Enjoyment of food → OW/OB	< 0.001	< 0.001	0.569	< 0.001 to 0.001	-
	Laissez-faire → Surgency → Satiety responsiveness → OW/OB	< 0.001	0.001	0.501	−0.003 to 0.001	-
	Laissez-faire → Surgency → Slowness in eating → OW/OB	< 0.001	< 0.001	0.316	−0.002 to <0.001	-
	Laissez-faire → Effortful control → Food responsiveness → OW/OB	0.001	0.001	0.218	−0.001 to 0.005	-
	Laissez-faire → Effortful control → Enjoyment of food → OW/OB	< 0.001	< 0.001	0.418	−0.001 to <0.001	-
	Laissez-faire → Effortful control → Satiety responsiveness → OW/OB	0.003	0.004	0.315	−0.003 to 0.012	-
	Laissez-faire → Effortful control → Slowness in eating → OW/OB	< 0.001	< 0.001	0.223	< 0.001 to 0.002	-
	Laissez-faire → Food fussiness → OW/OB	0.010	0.009	0.199	−0.005 to 0.030	-
	Laissez-faire → Negative affectivity → Food fussiness → OW/OB	< 0.001	0.001	0.473	−0.001 to 0.002	-
	Laissez-faire → Surgency → Food fussiness → OW/OB	−0.001	0.001	0.343	−0.005 to 0.001	-
	Laissez-faire → Effortful control → Food fussiness → OW/OB	< 0.001	0.001	0.220	< 0.001 to 0.003	-
Total effect	Laissez-faire → OW/OB	0.069	0.047	0.151	−0.027 to 0.156	-

OW/OB, overweight and obesity; SE, standard error; BC 95% CI, bias-corrected 95% confidence interval. ^a^When *p* < 0.05, the path, *β*, SE, *p*, BC 95% CI and ratio (*100) Specific Indirect Effect to Total Effect are bolded. ^b^Ratio was calculated as 100 × (indirect effect (*β*)/total effect), where the total effect was the sum of the direct and indirect effects.

## Discussion

The novelty of this cross-sectional study lies in attempting to identify the pathways from feeding practices to childhood OW/OB through eating behaviors and temperament among children aged 6–23 months in the setting of low and middle-income countries (LMICs). Responsive feeding negatively predicted childhood OW/OB through the sequential mediation of higher effortful control and satiety responsiveness, and the sequential mediation of higher effortful control and lower food responsiveness, the sequential mediation of lower negative affectivity and food responsiveness, respectively. Restrictive feeding positively predicted childhood OW/OB through the sequential mediation of lower effortful control and satiety responsiveness, the sequential mediation of lower effortful control and higher food responsiveness, and the sequential mediation of higher surgency and lower food fussiness, respectively. Indulgent feeding positively predicted childhood OW/OB through the sequential mediation of higher surgency and food responsiveness.

Infant and young child with higher appetites and weight gains in the first 2 years of life, had a higher feeding frequency and were less sensitive to satiety cues (48). Responsive feeding promotes healthy weight gain by enhancing the development of self-regulation, highly controlling feeding that may disrupt the development of self-regulation in childhood and lead to the overconsumption of restricted foods (34). Responsive feeding is characterized by high responsivity and moderate restriction of diet quality and quantity (34), is associated with higher diet quality (49), lower weight status (50), and a reduced risk of obesity (51), compared with highly controlling feeding (52). In this study, Chinese mothers reported higher responsive feeding, consistent with previous study (3), directly predicted lower childhood OW/OB; and indirectly predicted lower childhood OW/OB through the sequential mediation of childhood eating behaviors and temperament. Responsive feeding encourages children to eat autonomously in response to physiological needs, encouraging self-regulation in eating and supporting cognitive, emotional, and social development in infants and young children (53). Children with responsive mothers were a lower proportion of those classified as OW/OB (54), and mothers were less likely to pressure children to eat or use food as a reward (50). Responsive mothers less frequently used food to soothe distressed children and their children had lower perceived emotional overeating, while mothers responding to distressed children in early life had implications for the development of maladaptive eating in later childhood (55). However, in this study, mothers did not report emotional overeating. Due to the escalating sociometric ratings and obesogenic environment over the past four decades in China, children are likely to develop obesogenic eating behaviors. In response, parents who engage in responsive feeding may improve childhood satiety responsiveness behavior to prevent childhood OW/OB (56).

Non-responsive feeding involving the restriction of food quantity and quality, and was found to be higher in Chinese mothers than in a diverse USA sample, it has been identified as a detrimental factor in appetite regulation among children (57). Restrictive feeding is associated with poorer self-regulation of appetite and limited consumption of fruits and vegetables (58). Fruit and vegetable consumption is related to obesity prevention, but restrictive feeding may result in later preference for restricted foods and increased consumption of these restricted foods when the restriction is removed, potentially increasing the risk of obesity over time (59). Restrictive feeding shows a positive association with food responsiveness behavior and a negative association with satiety responsiveness behavior, the findings in this study consistent with Western developed countries (20). Restrictive feeding was positively correlated with food responsiveness (23), which may increase childhood desire to eat forbidden food (17). Restricting access to a palatable food has been found to increase energy intake in children (60), children may learn to respond to food cues rather than internal cues of hunger and satiety, and parents who adopt highly restrictive feeding may be associated with a higher risk of childhood OW/OB (61). This study showed restrictive feeding was not associated with food fussiness behavior in children, which is not in line with a UK study (62). Previous studies either did not found that higher restrictive feeding was associated with higher childhood OW/OB (63), or that higher restrictive feeding reduced the risk of weight gain (64). In this study, restrictive feeding was directly associated with higher childhood OW/OB, positively predicted childhood OW/OB through the sequential mediation of lower effortful control and satiety responsiveness, and the sequential mediation of lower effortful control and higher food responsiveness, respectively.

Pressuring feeding has been associated with lower intuitive eating and higher disordered eating behaviors in children (65). Previous studies either did not report an association between pressuring feeding and childhood OW/OB (66), or report the association of pressuring feeding with poorer diet quality (67), lower weight status (68), and lower childhood OW/OB (10). This study revealed that food avoidant behaviors, e.g., slowness in eating and food fussiness were mediators in the association between pressuring feeding and childhood OW/OB. These findings are consistent with studies in the UK and Nigeria, which found that pressuring feeding was associated with slowness in eating and satiety responsiveness behaviors (69). However, an Australian study did not report any relationship between pressuring feeding and food fussiness behavior (23). A Norwegian study showed that higher pressuring feeding, e.g., using food as a reward, predicted increased food approach behaviors, e.g., enjoyment of food (70). However, a Ethiopian study did not report an association between pressuring feeding and food approach behaviors, e.g., food responsiveness and enjoyment of food (17). In LMICs, where food scarcity and undernutrition were major threats to childhood survival in the past, feeding practices have evolved in response to these threats (71). Pressuring feeding, e.g., encouraging children to finish food is customary in LMICs cultures, this area has not received enough research and social attention, which may be why Chinese mothers in this study did not report their children engaging in emotional overeating behavior, as they may not distinguish emotional overeating from “big appetite” (17).

Laissez-faire feeding can be conceptualized as the opposite of monitoring (72), previous studies either report that higher monitoring was associated with less childhood overweight (73), or did not report this association (74). In this study, no pathways were identified from pressuring and laissez-faire feeding to childhood OW/OB through the sequential mediation of childhood eating behaviors and temperament. Indulgent feeding is significantly associated with higher consumption of unhealthy foods and lower nutrient intake in children (7), and higher childhood OW/OB (75). Children with indulgent parents were found to gain excess weight (76), Chinese mothers with lower indulgent feeding positively restricted the amount of unhealthy foods for their children (7), this study showed that Chinese mothers engaged in lower indulgent feeding, positively predicted childhood OW/OB through lower food fussiness. In a USA study, children with higher negative affectivity significantly experienced pressuring feeding, and food fussiness behavior (77).

Few studies have focused on the mediators of childhood temperament and eating behaviors in the relationship between feeding practices and childhood OW/OB. Previous studies reported that temperament was associated with feeding practices (78), childhood eating behaviors (79), and childhood OW/OB (9). The combination of feeding practices and temperament has been identified as important predictor in predicting childhood eating behaviors and the risk of OW/OB (4). In similar environments, inherent characteristic of children can be influenced differently, e.g., infants with a highly negative temperament may respond distinctively to parental soothing, potentially impacting early childhood development depending on feeding practices (4). In the development of childhood temperament related to eating behaviors, the process may occur within feeding practices where parents who tend to one type of feeding practices, e.g., restrictive feeding interacts with emotionally reactive children, may forgo restriction in favor of calming emotional children (4). This study did not report any effect of restriction feeding on negative affectivity and surgency. Feeding practices may be based on childhood temperament, e.g., parents adapted the restriction feeding to soothe or reward foods to children with negative temperament; and inhibited children were more likely to develop food neophobia, especially when their parents reported pressuring feeding (80). However, this study did not report any effect of restrictive feeding on negative affectivity. Parents may also soothe infants using food with higher surgency (higher activity and approach to novelty or reward) (9). This study did not report the sequential mediation from pressuring feeding to childhood eating behaviors and OW/OB.

Infants exhibit effortful control, characterized by the ability to initiate, maintain, and cease activities and awareness of rules (4). Attentional behaviors, e.g., focusing and sustained attention, form the foundation of effortful control (81), and the emergence of temperament-based effortful control (82) is significantly associated with the development of childhood OW/OB (83). In this study, responsive and restrictive feedings negatively predicted childhood OW/OB through effortful control, food responsiveness and satiety responsiveness. Negative affectivity of temperament was significantly associated with neophobic eating behaviors (80), and the risk of obesity (84). Difficult temperament is reliable measure of negative emotionality (4), and indulgent feeding is directly related to childhood food fussiness, mediated by negative affectivity (35). Anger reactivity is the emotion responsible for the relationship with overeating, while fear reactivity is highly related to undereating (4). Higher negative affectivity was related to higher pressuring feeding and childhood food fussiness (77). Fear reactivity is significantly related to food neophobia (4), and reactivity and regulation affects childhood OW/OB (85). Thus, temperament is indirectly associated with feeding practices, childhood eating behaviors and OW/OB (4). Negative affectivity predicts food approach behaviors, e.g., emotional overeating, and food avoidant behaviors, e.g., food fussiness and emotional undereating (86). This study reported that responsive feeding negatively predicted childhood OW/OB through the sequential mediation of negative affectivity and enjoyment of food. The frequency of consumption of sugar-rich foods and drinks (SFD) depended on childhood negative affectivity, children with higher negative affectivity consumed higher SFD when plenty of these foods were available (87). Children with effortful control were positively associated with self-regulated satiety responsiveness, and negatively associated with childhood OW/OB (86). This study reported that only responsive feeding was directly associated with the effect of childhood negative affectivity behaviors on OW/OB through food responsiveness behavior. This study did not report surgency behavior as a mediator in the pathways from feeding practices to childhood behaviors and OW/OB.

### Strength and recommendations

To the best of our knowledge, this is the first study in a LMIC to identify the pathways from maternal feeding practices to childhood OW/OB through the mediators of childhood temperament and eating behaviors. The SEM combines confirmatory factor analysis (CFA) and path analysis estimating latent variables, attributes the relationship among variables. The SEM approach including sociodemographic variables as covariates is valuable in providing methodological challenges in estimating direct and indirect effects through multiple pathways to propose targeted interventions. This study provides relevant evidence regarding maternal feeding practices, and the mediators of childhood temperament and eating behaviors, can guide intervention strategies aimed at preventing childhood OW/OB. The research design provided a varied sample including lower socioeconomic status (SES) families, e.g., mothers with lower education. The study sample primarily consisted of unemployed mothers (55%) who did not regularly take their children for health checkups, which indicates inadequate data collection on infant and young child aged 0–3 years and their families, particularly in LMICs, allowing governments to avoid the responsibilities for progressing of infant and young child feeding. The participants were sampled from a population not studied well in the same field. This sudy combined physical measurements of BMIz to determine the childhood OW/OB status. Policymakers in LMICs should prioritize improving maternal feeding practices through childhood temperament and eating behaviors to prevent childhood OW/OB. Such action is required to adopt new or enhanced national data collections and analytical approaches, e.g., incorporating infant and young child feeding practices into national health systems, a healthcare professional training on infant and young child feeding. Recommendations for using fiscal policy to make larger investments in maternal, infant, and young child health and nutrition are warranted. Longitudinal studies with large and representative population samples are essential to further fully elucidate the pathways linking maternal feeding practices and childhood OW/OB regrading childhood eating behaviors and temperament when designing mitigation intervention strategies.

### Limitations

This cross-sectional study is limited for the interpretation of the causality, and maternal feeding practices, childhood eating behaviors and temperament are dynamic and changing landscapes. Feeding practices, childhood temperament, and eating behaviors were measured using primarily maternal report data, which are of particular concern (4), but the maternal feeding practices may well reflect maternal concern about the infant and young child being over-or underweight. There is one measurement of BMIz, the conclusions maybe shallow and overstated. This study did not collect other factors that can be the participation in the feeding activity as confounding effects, e.g., grandparents’ involvement in feeding practices. This study had a unidirectional perspective, maternal feeding is a dynamic process to which both child and mother contribute (88), further studies of bidirectional relationships between maternal feeding practices and childhood temperament, eating behaviors and OW/OB are required.

## Conclusion

Feeding practices negatively predicted childhood OW/OB through the mediators of childhood temperament and eating behaviors in children aged 6–23 month, which may be beneficial to identify early interventions of healthy weight trajectories. This study could help governments agencies, policymakers, and healthcare workers to establish optimal intervention programs targeting maternal feeding practices through childhood eating behaviors and temperament to prevent childhood OW/OB in LMICs.

## Data availability statement

The data analyzed in this study is subject to the following licenses/restrictions: availability of data and materials. The datasets generated and analyzed during the current study are not publicly available due to original consent, but are available from the corresponding author upon reasonable request. Requests to access these datasets should be directed to XZ, xiaoning.zhang22@outlook.com.

## Ethics statement

The studies involving humans were approved by Xuzhou Medical University ethics committee. The studies were conducted in accordance with the local legislation and institutional requirements. Written informed consent for participation in this study was provided by the participants’ legal guardians/next of kin.

## Author contributions

XZ designed and supervised the study, prepared the manuscript, developed the conceptual framework, analyzed the data, interpreted the results, and wrote the original and final manuscript. QZ developed the conceptual framework, collected, analyzed, and interpreted the results. WL and SW aided in interpreting the results. NV drafted the final manuscript. JC managed and supervised the study. All authors have read and agreed to the published version of the manuscript.

## Funding

This study was funded by the Key Research Development project of Xuzhou (KC22295), and Hangzhou Normal University. This project was also supported by the Scientific Research Fund of Zhejiang Provincial Education Department (y202351682). The funding bodies were not involved in the study design, data collection, or data analysis, or in the writing of the manuscript.

## Conflict of interest

The authors declare that the research was conducted in the absence of any commercial or financial relationships that could be construed as a potential conflict of interest.

## Publisher’s note

All claims expressed in this article are solely those of the authors and do not necessarily represent those of their affiliated organizations, or those of the publisher, the editors and the reviewers. Any product that may be evaluated in this article, or claim that may be made by its manufacturer, is not guaranteed or endorsed by the publisher.

## Figures and Tables

**Table:** 

AGFI	Adjusted Goodness-of-fit Index
BMI	Body mass index
BMIz	Body mass index *z*-score
CEBQ-T	The Child Eating Behavior Questionnaire for toddler
*CF*	Complementary feeding
CI	Confidence interval
OW/OB	Childhood overweight and obesity
EF	Enjoyment of food
EOE	Emotional overeating
FR	Food responsiveness
FF	Food fussiness
GFI	Goodness of fit index
IBQ-RSF	The short form of Infant Behavior Questionnaire Revised
IFSQ	The Infant Feeding Style Questionnaire
IYCF	Infant and young child feeding
LMIC	Low- and middle-income country
Revised IBQ-RSF	The short form of Infant Behavior Questionnaire Revised
RMSEA	Root-mean-square error of approximation
SEM	The structural equation modeling
SR	Satiety responsiveness
SRMR	Standardized root-mean-square residual
