# Supplementary material for: Sequential mediation of early temperament and eating behaviors in the pathways from feeding practices to childhood overweight and obesity
Source: Front Public Health. 2023 Sep 11;11:1122645. doi: 10.3389/fpubh.2023.1122645 (PMC10520502; doi:10.3389/fpubh.2023.1122645)
Supplement: Supplementary file 1 [file Table_1.PDF]

Infant Feeding Style Questionnaire (IFSQ)

Behaviors are scored: ascending 1-never, 2-seldom, 3-half of the time, 4-most of the time, and 5-always; descending 5-never, 4- seldom, 3-half of the time, 2-most of the time, 1-always.

Belief items are scored: ascending 1-disagree, 2-slightly disagree, 3-neutral, 4-slightly agree, and 5-agree; descending 5-disagree, 4- slightly disagree, 3-neutral, 2-slightly agree, 1-agree.

Feeding Style		Item Description	Scoring
LAISSEZ-FAIRE			
Attention		*Behavior items*	
	LF1	When (name of child) has/had a bottle, I prop/propped it up	Ascending
	LF2	(Child) watches TV while eating	Ascending
	LF3	I watch TV while feeding (child)	Ascending
		Belief items	
	LF4	I think it is okay to prop an infant's bottle	Ascending
	LF5	It's okay for a toddler to walk around while eating as long as s/he eats	Ascending
Diet quality		*Behavior items*	
	LF6	I keep track of what food (child) eats	Descending
	LF7	I keep track of how much food (child) eats	Descending
	LF8	I make sure (child) does not eat sugary food like candy, ice cream, cakes or cookies	Descending
	LF9	I make sure (child) does not eat junk food like potato chips, Doritos and cheese puffs	Descending
		Belief items	
	LF10	A toddler should be able to eat whatever s/he wants for snacks	Ascending
	LF11	A toddler should be able to eat whatever s/he wants when eating out at a restaurant	Ascending
PRESSURING			
Finishing		*Behavior items*	
	PR1	Try to get (child) to finish his/her food	Ascending
	PR2	If (child) seems full, encourage to finish anyway	Ascending
	PR3	Try to get (child) to finish breastmilk or formula	Ascending
	PR4	Try to get (child) to eat even if not hungry	Ascending
	PR5	Insist re-try new food refused at same meal	Ascending
	PR6	Praise after each bite to encourage finish food	Ascending
		Belief Items	
	PR7	Important for toddler finish all food on his/her plate	Ascending
	PR8	Important for infant finish all milk in his/her bottle	Ascending
Cereal		*Behavior items*	
	PR11	Give/gave (child) cereal in the bottle	Ascending
		Belief items	
	PR12	Cereal in bottle helps infant sleep thru the night	Ascending
	PR13	Putting cereal in bottle good b/c helps infant feel full	Ascending
	PR14	An infant <6 mo needs more than formula or breastmilk to be full	Ascending
	PR15	An infant <6 mo needs more than formula or breastmilk to sleep through the night	Ascending

Soothing		*Behavior items*	
	PR16	When (child) cries, immediately feed him/her	Ascending
		Belief items	
	PR17	Best way to make infant stop crying is to feed	Ascending
	PR18	Best way to make toddler stop crying is to feed	Ascending
	PR19	When infant cries, usually means s/he needs to be fed	Ascending
RESTRICTIVE			
Amount		*Behavior items*	
	RS1	I carefully control how much (child) eats	Ascending
	RS2	I am very careful not to feed (child) too much	Ascending
		Belief Items	
	RS3	Important parent has rules re: how much toddler eats	Ascending
	RS4	Important parent decides how much infant should eat	Ascending
Diet Quality		*Behavior items*	
	RS5	I let (child) eat fast food	Descending
	RS6	I let (child) eat junk food	Descending
		Belief items	
	RS7	A toddler should never eat fast food	Ascending
	RS8	An infant should never eat fast food	Ascending
	RS9	A toddler should never eat sugary food like cookies	Ascending
	RS10	A toddler should never eat junk food like chips	Ascending
	RS11	A toddler should only eat healthy food	Ascending
RESPONSIVE			
Satiety		*Behavior items*	
	RP1	(Child) lets me know when s/he is full	Ascending
	RP2	(Child) lets me knows when s/he is hungry	Ascending
	RP3	I let (child) decide how much to eat	Ascending
	RP4	I pay attention when (child) seems to be telling me that s/he is full or hungry	Ascending
	RP5	I allow (child) to eat when s/he is hungry	Ascending
		Belief Items	
	RP6	Child knows when s/he is full	Ascending
	RP7	Child knows when hungry, needs to eat	Ascending
Attention		*Behavior items*	
	RP8	Talk to (child) to encourage to drink formula/breastmilk	Ascending
	RP9	Talk to (child) to encourage him/her to eat	Ascending
	RP10	Show (child) how to eat by taking a bite or pretending to	Ascending
	RP11	I will retry new foods if they are rejected at first	Ascending
		Belief items	
	RP12	Important to help or encourage a toddler to eat	Ascending
INDULGENCE			
Permissive		*Behavior items*	
	ID1	Allow child watch TV while eating if s/he wants	Ascending
	ID2	Allow child to eat fast food if s/he wants[c]	Ascending
	ID3	Allow child to drink sugared drinks/soda if s/he wants	Ascending
	ID4	Allow child to eat desserts/sweets if s/he wants	Ascending
		Belief Items	
	ID5	Toddlers should be allowed to watch TV while eating if they want	Ascending
	ID6	Toddlers should be allowed to eat fast food if they want	Ascending

	ID7	Toddlers should be allowed to drink sugared drinks/soda if they want	Ascending
	ID8	Toddlers should be allowed to eat desserts/sweets if they want	Ascending
Coaxing		*Behavior items*	
	ID9	Allow child watch TV while eating to make sure s/he gets enough	Ascending
	ID10	Allow child to eat fast food to make sure s/he gets enough	Ascending
	ID11	Allow child to drink sugared drinks/soda to make sure s/he gets enough	Ascending
	ID12	Allow child to eat desserts/sweets to make sure s/he gets enough	Ascending
		-*Belief Items*	
	ID13	Toddlers should be allowed to watch TV while eating to make sure they get enough	Ascending
	ID14	Toddlers should be allowed to eat fast food to make sure they get enough	Ascending
	ID15	Toddlers should be allowed to drink sugared drinks/soda to make sure they get enough	Ascending
	ID16	Toddlers should be allowed to eat desserts/sweets to make sure they get enough	Ascending
Soothing		*Behavior items*	
	ID17	Allow child watch tv while eating to keep him/her from crying	Ascending
	ID18	Allow child to eat fast food to keep him/her from crying	Ascending
	ID19	Allow child to drink sugared drinks/soda to keep him/her from crying	Ascending
	ID20	Allow child to eat desserts/sweets to keep him/her from crying	Ascending
		Belief Items	
	ID21	Toddlers should be allowed to watch tv while eating to keep them from crying	Ascending
	ID22	Toddlers should be allowed to eat fast food to keep them from crying	Ascending
	ID23	Toddlers should be allowed to drink sugared drinks/soda to keep them from crying	Ascending
	ID24	Toddlers should be allowed to eat desserts/sweets to keep them from crying	Ascending
Pampering		*Behavior items*	
	ID25	Allow child watch tv while eating to keep him/her happy	Ascending
	ID26	Allow child to eat fast food to keep him/her happy	Ascending
	ID27	Allow child to drink sugared drinks/soda to keep him/her happy	Ascending
	ID28	Allow child to eat desserts/sweets to keep him/her happy	Ascending
		Belief Items	
	ID29	Toddlers should be allowed to watch tv while eating to keep them happy	Ascending
	ID30	Toddlers should be allowed to eat fast food to keep them happy	Ascending
	ID31	Toddlers should be allowed to drink sugared drinks/soda to keep them happy	Ascending
	ID32	Toddlers should be allowed to eat desserts/sweets to keep them happy	Ascending

Infant Behavior Questionnaire - Revised

Subject No. _______________

Today's Date _______________

Sex of Child _______________

Date of Baby's Birth ______ ____ _____
month. day year

Age of Child _____ _____
mos. weeks

INSTRUCTIONS:
Please read carefully before starting:

As you read each description of the baby's behavior below, please indicate how often the baby did this during the LAST WEEK (the past seven days) by circling one of the numbers in the left column. These numbers indicate how often you observed the behavior described during the last week.

(1) Never	(2) Very Rarely	(3) Less Than Half the Time	(4) About Half the Time	(5) More Than Half the Time	(6) Almost Always	(7) Always	(X) Does Not Apply

The "Does Not Apply" (X) column is used when you did not see the baby in the situation described during the last week. For example, if the situation mentions the baby having to wait for food or liquids and there was no time during the last week when the baby had to wait, circle the (X) column. "Does Not Apply" is different from "Never" (1). "Never" is used when you saw the baby in the situation but the baby never engaged in the behavior listed during the last week. For example, if the baby did have to wait for food or liquids at least once but never cried loudly while waiting, circle the (1) column.

Please be sure to circle a number for <u>every</u> item.

(1) Never	(2) Very Rarely	(3) Less Than Half the Time	(4) About Half the Time	(5) More Than Half the Time	(6) Almost Always	(7) Always	(X) Does Not Apply

One Week Time Span

How often did your baby:

1 2 3 4 5 6 7 X . . . . (1) make talking sounds when s/he was ready for more food?

1 2 3 4 5 6 7 X . . . . (2) seem angry (crying and fussing) when you left her/him in the crib?

1 2 3 4 5 6 7 X . . . . (3) seem contented when left in the crib?

1 2 3 4 5 6 7 X . . . . (4) cry or fuss before going to sleep for naps?

1 2 3 4 5 6 7 X . . . . (5) look at pictures in books and/or magazines for 5 minutes or longer at a time?

1 2 3 4 5 6 7 X . . . . (6) stare at a mobile, crib bumper or picture for 5 minutes or longer?

1 2 3 4 5 6 7 X . . . . (7) play with one toy or object for 5-10 minutes?

1 2 3 4 5 6 7 X . . . . (8) play with one toy or object for 10 minutes or longer?

1 2 3 4 5 6 7 X . . . . (9) laugh aloud in play?

1 2 3 4 5 6 7 X . . . . (10) repeat the same movement with an object for 2 minutes or longer (e.g., putting a block in a cup, kicking or hitting a mobile)?

1 2 3 4 5 6 7 X . . . . (11) smile or laugh after accomplishing something (e.g., stacking blocks, etc.)?

1 2 3 4 5 6 7 X . . . . (12) smile or laugh when given a toy?

1 2 3 4 5 6 7 X . . . . (13) enjoy being read to?

1 2 3 4 5 6 7 X . . . . (14) enjoy hearing the sound of words, as in nursery rhymes?

1 2 3 4 5 6 7 X . . . . (15) enjoy gentle rhythmic activities, such as rocking or swaying?

1 2 3 4 5 6 7 X . . . . (16) enjoy being tickled by you or someone else in your family?

1 2 3 4 5 6 7 X . . . . (17) enjoy the feel of soft blankets ?

1 2 3 4 5 6 7 X . . . . (18) enjoy being rolled up in a warm blanket?

(1) Never	(2) Very Rarely	(3) Less Than Half the Time	(4) About Half the Time	(5) More Than Half the Time	(6) Almost Always	(7) Always	(X) Does Not Apply

1 2 3 4 5 6 7 X . . . . (19) enjoy listening to a musical toy in a crib?

1 2 3 4 5 6 7 X . . . . (20) look up from playing when the telephone rang?

1 2 3 4 5 6 7 X . . . . (21) protest being placed in a confining place (infant seat, play pen, car seat, etc)?

1 2 3 4 5 6 7 X . . . . (22) startle at a sudden change in body position (for example, when moved suddenly)?

1 2 3 4 5 6 7 X . . . . (23) move quickly toward new objects?

1 2 3 4 5 6 7 X . . . . (24) show a strong desire for something s/he wanted?

1 2 3 4 5 6 7 X . . . . (25) watch adults performing household activities (e.g., cooking, etc.) for more than 5 minutes?

1 2 3 4 5 6 7 X . . . . (26) squeal or shout when excited?

1 2 3 4 5 6 7 X . . . . (27) notice low-pitched noises (e.g. air conditioner, heating system, or refrigerator running or starting up)?

1 2 3 4 5 6 7 X . . . . (28) notice a change in light when a cloud passed over the sun?

1 2 3 4 5 6 7 X . . . . (29) notice the sound of an airplane passing overhead?

1 2 3 4 5 6 7 X . . . . (30) notice a bird or a squirrel up in a tree?

1 2 3 4 5 6 7 X . . . . (31) notice fabrics with scratchy texture (e.g., wool)?

1 2 3 4 5 6 7 X . . . . (32) appear sad for no apparent reason?

During feeding, how often did the baby:

1 2 3 4 5 6 7 X . . . . (33) lie or sit quietly?

1 2 3 4 5 6 7 X . . . . (34) squirm or kick?

1 2 3 4 5 6 7 X . . . . (35) wave his/her arms?

(1) Never	(2) Very Rarely	(3) Less Than Half the Time	(4) About Half the Time	(5) More Than Half the Time	(6) Almost Always	(7) Always	(X) Does Not Apply

When going to sleep at night, how often did your baby:

1 2 3 4 5 6 7 X . . . . (36) fall asleep within 10 minutes?

1 2 3 4 5 6 7 X . . . . (37) have a hard time settling down to sleep?

1 2 3 4 5 6 7 X . . . . (38) settle down to sleep easily?

When being dressed or undressed during the last week, how often did the baby:

1 2 3 4 5 6 7 X . . . . (39) squirm and/or try to roll away?

1 2 3 4 5 6 7 X . . . . (40) smile or laugh?

1 2 3 4 5 6 7 X . . . . (41) coo or vocalize?

When put into the bath water, how often did the baby:

1 2 3 4 5 6 7 X . . . . (42) smile?

1 2 3 4 5 6 7 X . . . . (43) laugh?

When tossed around playfully how often did the baby:

1 2 3 4 5 6 7 X . . . . (44) smile?

1 2 3 4 5 6 7 X . . . . (45) laugh?

During a peekaboo game, how often did the baby:

1 2 3 4 5 6 7 X . . . . (46) smile?

1 2 3 4 5 6 7 X . . . . (47) laugh?

How often did your baby enjoy bouncing up and down:

1 2 3 4 5 6 7 X . . . . (48) while on your lap?

1 2 3 4 5 6 7 X . . . . (49) on an object, such as a bed, bouncer chair, or toy?

When being held, how often did the baby:

1 2 3 4 5 6 7 X . . . . (50) pull away or kick?

1 2 3 4 5 6 7 X . . . . (51) seem to enjoy him/herself?

(1) Never	(2) Very Rarely	(3) Less Than Half the Time	(4) About Half the Time	(5) More Than Half the Time	(6) Almost Always	(7) Always	(X) Does Not Apply

When the baby wanted something, how often did s/he:

1 2 3 4 5 6 7 X . . . . (52) become upset when s/he could not get what s/he wanted?

1 2 3 4 5 6 7 X . . . . (53) have tantrums (crying, screaming, face red, etc.) when s/he did not get what s/he wanted?

When placed in an infant seat or car seat, how often did the baby:

1 2 3 4 5 6 7 X . . . . (54) wave arms and kick?

1 2 3 4 5 6 7 X . . . . (55) squirm and turn body?

How often did your baby make talking sounds when:

1 2 3 4 5 6 7 X . . . . (56) riding in a car?

1 2 3 4 5 6 7 X . . . . (57) riding in a shopping cart?

1 2 3 4 5 6 7 X . . . . (58) you talked to her/him?

When rocked or hugged, in the last week, how often did your baby:

1 2 3 4 5 6 7 X . . . . (59) seem to enjoy her/himself?

1 2 3 4 5 6 7 X . . . . (60) seem eager to get away?

1 2 3 4 5 6 7 X . . . . (61) While being fed in your lap, how often did the baby seem eager to get away as soon as the feeding was over?

1 2 3 4 5 6 7 X . . . . (62) After sleeping, how often did the baby cry if someone didn't come within a few minutes?

1 2 3 4 5 6 7 X . . . . (63) When put down for a nap, how often did your baby settle down quickly?

1 2 3 4 5 6 7 X . . . . (64) When it was time for bed or a nap and your baby did not want to go, how often did s/he whimper or sob?

1 2 3 4 5 6 7 X . . . . (65) When face was washed, how often did the baby smile or laugh?

1 2 3 4 5 6 7 X . . . . (66) When hair was washed, how often did the baby vocalize?

1 2 3 4 5 6 7 X . . . . (67) When playing quietly with one of her/his favorite toys, how often did your baby enjoy lying in the crib for more than 5 minutes?

(1) Never	(2) Very Rarely	(3) Less Than Half the Time	(4) About Half the Time	(5) More Than Half the Time	(6) Almost Always	(7) Always	(X) Does Not Apply

1 2 3 4 5 6 7 X . . . . (68) When your baby saw a toy s/he wanted, how often did s/he get very excited about getting it?

1 2 3 4 5 6 7 X . . . . (69) When given a new toy, how often did your baby immediately go after it?

1 2 3 4 5 6 7 X . . . . (70) When placed on his/her back, how often did the baby squirm and/or turn body?

1 2 3 4 5 6 7 X . . . . (71) When frustrated with something, how often did your baby calm down within 5 minutes?

1 2 3 4 5 6 7 X . . . . (72) When your baby was upset about something, how often did s/he stay upset for up to 20 minutes or longer?

1 2 3 4 5 6 7 X . . . . (73) When being carried, how often did your baby push against you until put down?

1 2 3 4 5 6 7 X . . . . (74) When tired, how often did your baby show distress?

1 2 3 4 5 6 7 X . . . . (75) At the end of an exciting day, how often did your baby become tearful?

Two Week Time Span

When introduced to an unfamiliar adult, how often did the baby:

1 2 3 4 5 6 7 X . . . . (76) cling to a parent?

1 2 3 4 5 6 7 X . . . . (77) refuse to go to the unfamiliar person?

1 2 3 4 5 6 7 X . . . . (78) never “warm up” to the unfamiliar adult?

When you were busy with another activity and your baby was not able to get your attention, how often did s/he:

1 2 3 4 5 6 7 X . . . . (79) become sad?

1 2 3 4 5 6 7 X . . . . (80) cry?

When singing or talking to your baby, how often did s/he:

1 2 3 4 5 6 7 X . . . . (81) soothe immediately?

1 2 3 4 5 6 7 X . . . . (82) take more than 10 minutes to soothe?

(1) Never	(2) Very Rarely	(3) Less Than Half the Time	(4) About Half the Time	(5) More Than Half the Time	(6) Almost Always	(7) Always	(X) Does Not Apply

When showing the baby something to look at, how often did s/he:

1 2 3 4 5 6 7 X . . . . (83) soothe immediately?

1 2 3 4 5 6 7 X . . . . (84) take more than 10 minutes to soothe?

When patting or gently rubbing some part of the baby’s body, how often did s/he:

1 2 3 4 5 6 7 X . . . . (85) soothe immediately?

1 2 3 4 5 6 7 X . . . . (86) take more than 10 minutes to soothe?

1 2 3 4 5 6 7 X . . . . (87) When in the presence of several unfamiliar adults, how often did the baby continue to be upset for 10 minutes or longer?

1 2 3 4 5 6 7 X . . . . (88) When visiting a new place, how often did the baby get excited about exploring new surroundings?

1 2 3 4 5 6 7 X . . . . (89) When an unfamiliar adult came to your home or apartment, how often did your baby cry when the visitor attempted to pick her/him up?

1 2 3 4 5 6 7 X . . . . (90) When familiar relatives/friends came to visit, how often did your
baby get excited?

1 2 3 4 5 6 7 X . . . . (91) When rocking your baby, how often did s/he take more than 10 minutes to soothe?

Early Childhood Behavior Questionnaire – Short Form

Child's name: ______________________ Child's birthdate: Mo:____ Day:____ Yr:____

Today's date: Month:____ Day:____ Yr:____ Child's age: ______Yrs, ______Months

Relation to child: ______________________ Sex of child (circle one): Male Female

INSTRUCTIONS: **Please read carefully before starting**.

As you read each description of the child's behavior below, please indicate how often the child did this during the last two weeks by circling one of the numbers in the right column. These numbers indicate how often you observed the behavior described during the last two weeks.

never	very rarely	less than half the time	about half the time	more than half the time	almost always	always	does not apply
**1**	**2**	**3**	**4**	**5**	**6**	**7**	**NA**

The "Does Not Apply" column (NA) is used when you did not see the child in the situation described during the last two weeks. For example, if the situation mentions the child going to the doctor and there was no time during the last two weeks when the child went to the doctor, circle the (NA) column. "Does Not Apply" (NA) is different from "NEVER" (1). "Never" is used when you saw the child in the situation but the child never engaged in the behavior mentioned in the last two weeks. Please be sure to circle a number or NA for every item.

When told that it was time for bed or a nap, how often did your child

1. get irritable? 1 2 3 4 5 6 7 NA

When approached by an unfamiliar person in a public place (for example, the grocery store), how often did your child

2. pull back and avoid the person? 1 2 3 4 5 6 7 NA
3. cling to a parent? 1 2 3 4 5 6 7 NA

During everyday activities, how often did your child

4. tap or drum with fingers on tables or other objects? 1 2 3 4 5 6 7 NA
5. become uncomfortable when his/her socks were not aligned properly on his/her feet? 1 2 3 4 5 6 7 NA
6. become distressed when his/her hands were dirty and/or sticky? 1 2 3 4 5 6 7 NA
7. notice low-pitched noises such as the air-conditioner, heater, or refrigerator running or starting up? 1 2 3 4 5 6 7 NA
8. blink a lot? 1 2 3 4 5 6 7 NA

While playing outdoors, how often did your child

9. enjoy sitting quietly in the sunshine? 1 2 3 4 5 6 7 NA
10. look immediately when you pointed at something? 1 2 3 4 5 6 7 NA
11. choose to take chances for the fun and excitement of it? 1 2 3 4 5 6 7 NA
12. seem to be one of the most active children? 1 2 3 4 5 6 7 NA

When s/he was carried, how often did your child

13. push against you until put down? 1 2 3 4 5 6 7 NA
14. snuggle up next to you? 1 2 3 4 5 6 7 NA

While having trouble completing a task (e.g., building, drawing, dressing), how often did your child

15. get easily irritated? 1 2 3 4 5 6 7 NA

When a familiar child came to your home, how often did your child

16. seek out the company of the child? 1 2 3 4 5 6 7 NA

When offered a choice of activities, how often did your child

17. stop and think before deciding?	1	2	3	4	5	6	7	NA
18. decide what to do very quickly and go after it?	1	2	3	4	5	6	7	NA

When asked NOT to, how often did your child

19. touch an attractive item (such as an ornament) anyway?	1	2	3	4	5	6	7	NA

During daily or evening quiet time with you and your child, how often did your child

20. enjoy just being quietly sung to?	1	2	3	4	5	6	7	NA
21. smile at the sound of words, as in nursery rhymes?	1	2	3	4	5	6	7	NA
22. enjoy just being talked to?	1	2	3	4	5	6	7	NA
23. enjoy rhythmic activities, such as rocking or swaying?	1	2	3	4	5	6	7	NA
24. want to be cuddled?	1	2	3	4	5	6	7	NA

While at home, how often did your child

25. show fear at a loud sound (blender, vacuum cleaner, etc.)?	1	2	3	4	5	6	7	NA
26. seem afraid of the dark?	1	2	3	4	5	6	7	NA

While bathing, how often did your child

27. sit quietly?	1	2	3	4	5	6	7	NA

When s/he was upset, how often did your child

28. change to feeling better within a few minutes?	1	2	3	4	5	6	7	NA

When engaged in play with his/her favorite toy, how often did your child

29. play for more than 10 minutes?	1	2	3	4	5	6	7	NA
30. continue to play while at the same time responding to your remarks or questions?	1	2	3	4	5	6	7	NA

When approaching unfamiliar children playing, how often did your child

31. watch rather than join in?	1	2	3	4	5	6	7	NA
32. seem uncomfortable?	1	2	3	4	5	6	7	NA

During everyday activities, how often did your child

33. move quickly from one place to another?	1	2	3	4	5	6	7	NA
34. notice the smoothness or roughness of objects s/he touched?	1	2	3	4	5	6	7	NA
35. become sad or blue for no apparent reason?	1	2	3	4	5	6	7	NA
36. pay attention to you right away when you called to him/her?	1	2	3	4	5	6	7	NA
37. seem to be disturbed by loud sounds?	1	2	3	4	5	6	7	NA
38. seem frightened for no apparent reason?	1	2	3	4	5	6	7	NA
39. seem to be irritated by tags in his/her clothes?	1	2	3	4	5	6	7	NA

After having been interrupted, how often did your child

40. return to a previous activity?	1	2	3	4	5	6	7	NA
41. have difficulty returning to the previous activity?	1	2	3	4	5	6	7	NA

When told that loved adults would visit, how often did your child

42. get very excited?	1	2	3	4	5	6	7	NA
43. become very happy?	1	2	3	4	5	6	7	NA

During quiet activities, such as reading a story, how often did your child

44. swing or tap his/her foot?	1	2	3	4	5	6	7	NA
45. fiddle with his/her hair, clothing, etc.?	1	2	3	4	5	6	7	NA
46. show repeated movements like squinting, hunching up the shoulders, or twitching the facial muscles?	1	2	3	4	5	6	7	NA

While playing indoors, how often did your child

47. like rough and rowdy games?	1	2	3	4	5	6	7 NA
48. enjoy playing boisterous games like 'chase'?	1	2	3	4	5	6	7 NA
49. enjoy vigorously jumping on the couch or bed?	1	2	3	4	5	6	7 NA

In situations where s/he is meeting new people, how often did your child

50. turn away?	1	2	3	4	5	6	7 NA

When being gently rocked or hugged, how often did your child

51. seem eager to get away?	1	2	3	4	5	6	7 NA

When encountering a new activity, how often did your child

52. sit on the sidelines and observe before joining in?	1	2	3	4	5	6	7 NA
53. get involved immediately?	1	2	3	4	5	6	7 NA

When visiting the home of a familiar child, how often did your child

54. engage in an activity with the child?	1	2	3	4	5	6	7 NA

When engaged in an activity requiring attention, such as building with blocks, how often did your child

55. move quickly to another activity?	1	2	3	4	5	6	7 NA
56. tire of the activity relatively quickly?	1	2	3	4	5	6	7 NA

While in a public place, how often did your child

57. seem uneasy about approaching an elevator or escalator?	1	2	3	4	5	6	7 NA
58. cry or show distress when approached by an unfamiliar animal?	1	2	3	4	5	6	7 NA
59. seem afraid of large, noisy vehicles?	1	2	3	4	5	6	7 NA
60. show fear when the caregiver stepped out of sight?	1	2	3	4	5	6	7 NA

When being dressed or undressed, how often did your child

61. squirm and try to get away?	1	2	3	4	5	6	7 NA
62. stay still?	1	2	3	4	5	6	7 NA

When told "no", how often did your child

63. stop the forbidden activity?	1	2	3	4	5	6	7 NA
64. become sadly tearful?	1	2	3	4	5	6	7 NA

Following an exciting activity or event, how often did your child

65. calm down quickly?	1	2	3	4	5	6	7 NA
66. have a hard time settling down?	1	2	3	4	5	6	7 NA
67. seem to feel down or blue?	1	2	3	4	5	6	7 NA

During everyday activities, how often did your child

68. easily shift attention from one activity to another?	1	2	3	4	5	6	7 NA
69. become bothered by sounds while in noisy environments?	1	2	3	4	5	6	7 NA
70. become bothered by scratchy materials like wool?	1	2	3	4	5	6	7 NA
71. notice changes in your appearance (such as wet hair, a hat, or jewelry)?	1	2	3	4	5	6	7 NA
72. appear to listen to even very quiet sounds?	1	2	3	4	5	6	7 NA
73. seem full of energy, even in the evening?	1	2	3	4	5	6	7 NA
74. become irritated when his/her clothes were tight?	1	2	3	4	5	6	7 NA

While playing indoors, how often did your child

75. run through the house?	1	2	3	4	5	6	7 NA
76. climb over furniture?	1	2	3	4	5	6	7 NA
77. enjoy activities such as being spun, etc.?	1	2	3	4	5	6	7 NA

When playing alone, how often did your child

78. become easily distracted?	1	2	3	4	5	6	7	NA
79. play with a set of objects for 5 minutes or longer at a time?	1	2	3	4	5	6	7	NA
80. tear materials close at hand?	1	2	3	4	5	6	7	NA

Before an exciting event (such as receiving a new toy), how often did your child

81. get very excited about getting it?	1	2	3	4	5	6	7	NA
82. remain pretty calm?	1	2	3	4	5	6	7	NA

When s/he asked for something and you said "no", how often did your child

83. become frustrated?	1	2	3	4	5	6	7	NA
84. protest with anger?	1	2	3	4	5	6	7	NA
85. have a temper tantrum?	1	2	3	4	5	6	7	NA
86. become sad?	1	2	3	4	5	6	7	NA

While playing or walking outdoors, how often did your child

87. notice sights or sounds (for example, wind chimes or water sprinklers)?	1	2	3	4	5	6	7	NA

When asked to wait for a desirable item (such as ice cream), how often did your child

88. go after it anyway?	1	2	3	4	5	6	7	NA
89. wait patiently?	1	2	3	4	5	6	7	NA

When being gently rocked, how often did your child

90. smile?	1	2	3	4	5	6	7	NA

When you removed something s/he should not have been playing with, how often did your child

91. become sad?	1	2	3	4	5	6	7	NA

While being held on your lap, how often did your child

92. seem to enjoy him/herself?	1	2	3	4	5	6	7	NA
93. mold to your body?	1	2	3	4	5	6	7	NA

When hearing about a future family outing (such as a trip to the playground), how often did your child

94. look forward to it?	1	2	3	4	5	6	7	NA

While looking at picture books on his/her own, how often did your child

95. become easily distracted?	1	2	3	4	5	6	7	NA

When a familiar adult, such as a relative or friend, visited your home, how often did your child

96. want to interact with the adult?	1	2	3	4	5	6	7	NA

When asked to do so, how often was your child able to

97. stop an ongoing activity?	1	2	3	4	5	6	7	NA
98. be careful with something breakable?	1	2	3	4	5	6	7	NA

When visiting a new place, how often did your child

99. not want to enter?	1	2	3	4	5	6	7	NA

While you were talking with someone else, how often did your child

100. easily switch attention from speaker to speaker?	1	2	3	4	5	6	7	NA

When you mildly criticized or corrected her/his behavior, how often did your child

101. get mad?	1	2	3	4	5	6	7	NA

<u>When s/he was upset, how often did your child</u>

102. cry for more than 3 minutes, even when being comforted?	1	2	3	4	5	6	7	NA
103. become easily soothed?	1	2	3	4	5	6	7	NA

<u>When you were busy, how often did your child</u>

104. find another activity to do when asked?	1	2	3	4	5	6	7	NA

<u>While playing outdoors, how often did your child</u>

105. want to jump from heights?	1	2	3	4	5	6	7	NA

<u>When around large gatherings of familiar adults or children, how often did your child</u>

106. enjoy playing with a number of different people?	1	2	3	4	5	6	7	NA

<u>When s/he was asked to share his/her toys, how often did your child</u>

107. become sad?	1	2	3	4	5	6	7	NA

Child Eating Behavior Questionnaire for toddler (CEBQ-T)

Items are scored: 1-never, 2-seldom, 3-half of the time, 4-most of the time, and 5-always.

Factor	Item Description
Food responsiveness	My child is always asking for food
	If allowed to, my child would eat too much
	Given the choice, my child would eat most of the time
	Even if my child is full up s/he finds room to eat his/her favourite food
Enjoyment of food	My child loves food
	My child is interested in food
	My child looks forward to mealtimes
	My child enjoys eating
Satiety responsiveness	My child has a big appetite
	My child leaves food on his/her plate or in the jar at the end of a meal
	My child gets full before his/her meal is finished
	My child gets full up easily
	My child cannot eat a meal if s/he has had a snack just before
Slowness in eating	My child finishes his/her meal quickly
	My child eats slowly
	My child takes more than 30 minutes to finish a meal
	My child eats more and more slowly during the course of a meal
Food fussiness	My child refuses new foods at first
	My child enjoys tasting new foods
	My child enjoys a wide variety of foods
	My child is difficult to please with meals
	My child is interested in tasting food s/he hasn’t tasted before
	My child decides that s/he doesn’t like a food, even without tasting it
